# Diverse Coordination Chemistry of the Whole Series Rare-Earth L-Lactates: Synthetic Features, Crystal Structure, and Application in Chemical Solution Deposition of Ln_2_O_3_ Thin Films

**DOI:** 10.3390/molecules28155896

**Published:** 2023-08-05

**Authors:** Ruslan Gashigullin, Mikhail Kendin, Irina Martynova, Dmitry Tsymbarenko

**Affiliations:** 1 Department of Materials Science, Lomonosov Moscow State University, Moscow 119991, Russia; 2 Department of Chemistry, Lomonosov Moscow State University, Moscow 119991, Russia

**Keywords:** lanthanide, coordination compound, coordination polymer, 2-hydroxypropanoate, lactic acid, X-ray diffraction, crystal structure, thin film, chemical solution deposition

## Abstract

Rare-earth (RE, Ln) carboxylates are widely studied as precursors of RE oxide-based nanomaterials; however, no systematic studies of RE L-lactates (HLact = 2-hydroxypropanoic acid) have been reported to date. In the present work, a profound structural investigation of RE L-lactates is carried out. A family of RE lactate complexes of the general formula LnLact_3_∙nH_2_O (Ln = La, Ce–Nd, Sm–Lu, Y; *n* = 2–3) are synthesized and characterized by CHN, TGA, and FTIR as well as by powder and single-crystal XRD methods.The existence of four novel structural types (**1-Ln**–**4-Ln**) is revealed. Compounds of the **1-Ln** type (Ln = La, Ce, Pr) exhibit a chain polymeric structure, whereas **2-Ln**–**4-Ln** compounds are molecular crystals consisting of dimeric (**2-Ln**; Ln = La, Ce–Nd) or monomeric (**3-Ln**–Ln = Sm–Lu, Y; **4-Ln**–Ln = Sm–Gd, Y) species. The crystal structures of **1-Ln**–**4-Ln** compounds are discussed in terms of their coordination geometry and supramolecular arrangement. Solutions of yttrium and lanthanum lactates with diethylenetriamine are applied for the chemical deposition of Y_2_O_3_ and La_2_O_3_ thin films.

## 1. Introduction

Coordination compounds based on aliphatic metal carboxylates attract significant scientific interest as versatile precursors of inorganic nanomaterials [1,2,3]. In particular, rare-earth (RE, Ln) carboxylates are widely used in the preparation of functional thin films (high-temperature superconductors, colossal magnetoresistance materials, high-k dielectrics, and many others) through the metal–organic chemical solution deposition (MOCSD) process [4,5,6,7]. The latter implies the preparation of a precursor solution and its deposition onto a substrate with subsequent multi-stage annealing that eventually yields the required oxide thin film [4]. The composition of the precursor solution plays an important role, as it determines crucial characteristics of the latter, namely, viscosity, adhesion to the substrate, and decomposition mechanism [8].

Regarding the solution deposition of RE oxide films, RE carboxylates represent a set of advantages over other classes of RE compounds (e.g., alkoxides and β-diketonates) due to their moisture resistance and low volatility [4,9], although non-volatile RE carboxylates are generally inapplicable in the chemical vapor deposition process, where metal β-diketonates are widely used [10,11,12]. On the other hand, the majority of RE carboxylates exhibit polymeric structures [13,14,15,16,17,18], making them poorly soluble in common organic solvents. The solubility of RE carboxylates can be significantly enhanced by the addition of ancillary chelating ligands (e.g., polyamines) and formation of highly soluble mixed-ligand complexes, which decompose at relatively low (<700 °C) temperatures to yield their respective RE oxides [7,9,19]. Among the various RE monocarboxylates, acetates and propionates are often used in the fabrication of oxide materials [20,21,22,23,24]; however, there are very few reports on the use of RE lactates (2-hydroxypropanoates) for this purpose. In particular, Xue et al. reported the preparation of Pb_0.98_La_0.02_(Zr_0.66_Sn_0.27_Ti_0.07_)O_3_ ceramics from a solution of the respective metal lactates [25]; however, no experiments on the deposition of RE sesquioxide thin films using lactate-based precursors have been published to date.

Although L-lactic acid (henceforth HLact) is widely used in modern chemistry and chemical technology, simple metal salts of HLact remain poorly studied. There are few reports on the synthesis and characterization of Zn [26,27,28], Co [29], Mn [30], Zr [31], and UO_2_^2+^ [32] lactates or the heterocationic and mixed-ligand compounds K_2_[{VO(O_2_)(Lact′)}_2_] [33], (NH_4_)_2_[Ti(Lact′)_3_] [34], (NEt_4_)_2_[VO_2_(Lact′)]_2_ and (Ph_3_P=N=PPh_3_)_2_[VO_2_(Lact′)]_2_ [35], (TMA)_3_[Mn_2_(Lact)(Lact′)_4_]∙H_2_O [36] (TMA = NMe_4_^+^), Zr(OH)Lact_3_ [31], (NH_4_)_4_[Ta_2_(Lact’)_4_(O_2_)_2_O]·3H_2_O [37], [Cu(Lact)Cl(dipyam)] (dipyam = 2,2′-dipyridylamine) [38], [Cu(Lact)(ClO_4_)(phen)]_4_ (phen = 1,10-phenanthroline) [39], [Ni_6_(Lact’)_2_(Lact)_2_Piv_6_(HPiv)_8_]·2HPiv (HPiv = pivalic acid) [40], [Cu(Lact)_2_(SS)]·4H_2_O, and [Cu_2_(Lact)_4_(SCS)_3_] (SS = 4,4′-dipyridyldisulfide; SCS = bis(4-pyridylthio)methane) [41] (here, Lact’ corresponds to the lactic acid dianion CH_3_CH(O^–^)COO^–^). Among RE lactates, only a few compounds have been synthesized and characterized to date. Namely, crystal structures of 2D-layered cationic {[Ln(H_2_O)_2_Lact_2_](ClO_4_)}_∞_ (Ln = Eu, Tb) [42,43] and molecular [Y(H_2_O)_2_Lact_3_] [44] compounds as well as mixed-ligand complexes of RE (L/D)-lactates with O- and N-donor ligands [45,46,47] have been reported. The main obstacle to the structural study of RE lactates lies in their tendency to form viscous supersaturated solutions or/and intergrown crystals rather than X-ray quality single crystals. Nevertheless, RE carboxylates are known to exhibit extreme structural diversity [13]; RE lactates seem to be an interesting system in this scope, as the presence of ancillary OH-groups near the carboxyl groups may yield unusual metal–ligand coordination modes, leading in turn to novel and unexpected supramolecular motifs. This feature can be illustrated on yttrium complexes with propionate and lactate ligands, which possess the same carbon chain structure while differing in the presence of an additional OH-group. Thus, yttrium propionate possesses a 2D-layered motif [18,48], whereas yttrium lactate exhibits a mononuclear structure, which is rather rare for RE carboxylates [44].

In the present work, we report a systematic X-ray diffraction study of RE lactates as well as their application in the chemical solution deposition of RE oxide thin films. A series of RE lactate complexes of the general formula LnLact_3_∙nH_2_O (Ln = La, Ce–Nd, Sm–Lu, Y; *n* = 2–3) have been synthesized and characterized by means of conventional chemical analyses (CHN, TGA, FTIR) as well as powder and single-crystal X-ray diffraction. Four structural types exhibiting different coordination environments of Ln^3+^ ions and different supramolecular arrangements have been revealed. Solutions of yttrium and lanthanum lactates with diethylenetriamine (DETA) were applied for the chemical deposition of Y_2_O_3_ and La_2_O_3_ thin films, respectively.

## 2. Results and Discussion

### 2.1. Synthesis of RE Lactates

**Figure 1:**
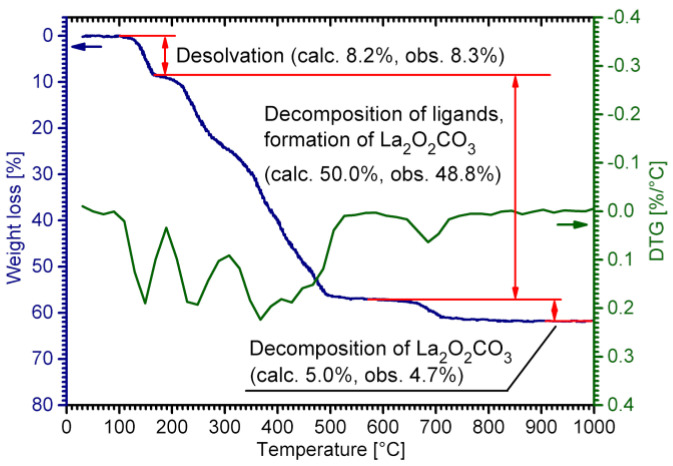
TG-DTG data for **1-La**. A full set of TG-DTG curves for RE lactates is provided in Figures S1–S4.

**Figure 2:**
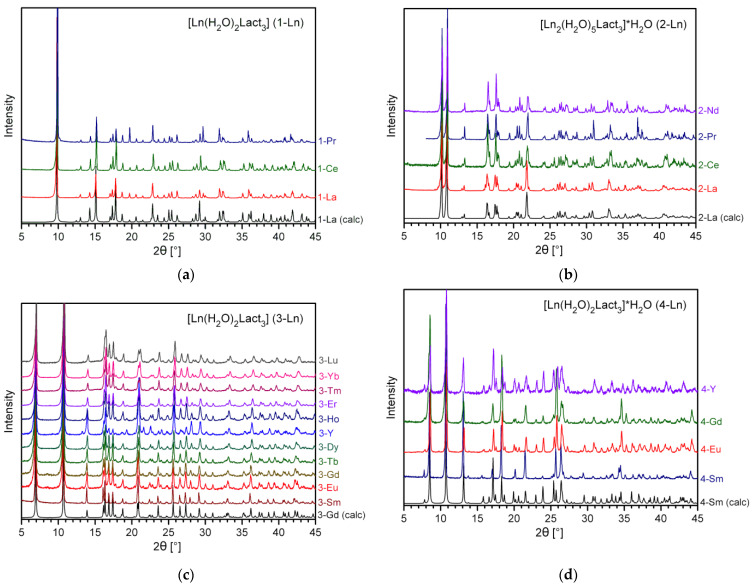
PXRD patterns for the samples of: (**a**) **1-Ln**; (**b**) **2-Ln**; (**c**) **3-Ln**; and (**d**) **4-Ln**. Experimental PXRD patterns are shown in comparison with the respective theoretical profiles calculated from the structure models at low (100–120 K) temperatures with room-temperature unit cell parameters (in the case of **2-La**, peak intensities estimated from the le Bail refinement were used to generate the theoretical profile).

In general, RE carboxylates are rather simple to synthesize; these compounds are commonly obtained via protolytic or ligand exchange reactions [49,50,51,52,53]. On the other hand, RE coordination compounds are known to exhibit structural flexibility; consequently, both the composition and structure of the synthesis product are highly sensitive to the preparation conditions. Regarding RE lactates, the hydrate number of the complex may easily vary depending on the synthesis temperature, concentration of water, and radius of the particular RE cation. An additional degree of freedom arises from the chirality of lactic acid and its anion; it is worth noting that the industrial HLact obtained by microbial fermentation consists of an almost pure L- (S, +) enantiomer. Thus, the presence of a racemic mixture of both enantiomers of the lactate ion or/and ancillary ions (even Na^+^ and ClO_4_^–^) in the initial solution can drastically change the reaction pathway, yielding heterocationic (e.g., {[LnNa(D-Lact)_2_(L-Lact)_2_]·2H_2_O}_∞_ − Ln = Sm, Eu [54]) or heteroanionic ({[Ln(H_2_O)_2_(L-Lact)_2_](ClO_4_)}_∞_ − Ln = Eu, Tb [42,43]) compounds. Nevertheless, the synthetic features of RE lactates have not been studied in detail yet, and the few papers dedicated to this issue represent only fragmentary and incomplete data. Therefore, we have carried out an extensive study on the preparation of RE lactates for the whole RE series (except for Sc and Pm) by means of different experimental procedures. According to the TGA (Figure 1 and Figures S1–S4) and FTIR data, all syntheses throughout Procedures I–III along with the room-temperature evaporation of mother liquors yielded the corresponding RE lactate complexes of the general formula LnLact_3_∙nH_2_O (Ln = La, Ce–Nd, Sm–Lu, Y; *n* = 2–3), which were divided into four types on the basis of powder and single-crystal XRD data (Figure 2): **1-Ln** ([Ln(H_2_O)_2_Lact_3_]_∞_; Ln = La, Ce, Pr), **2-Ln** ([Ln_2_(H_2_O)_5_Lact_6_]∙H_2_O; Ln = La, Ce, Pr, Nd), **3-Ln** ([Ln(H_2_O)_2_Lact_3_]; Ln = Sm–Lu, Y), and **4-Ln** ([Ln(H_2_O)_2_Lact_3_]∙H_2_O; Ln = Sm–Gd, Y). Regardless of the procedure (I–III) used, the phase composition of synthesis products is only affected by the crystallization and drying conditions.

In the case of light RE (La–Nd) lactates, the crystallization rate and temperature proved to be the major factors affecting the phase composition of the products. Indeed, fast evaporation of the solvent at room temperature (e.g., in air flow) yields the respective **1-Ln** complexes; X-ray quality single crystals of these can be prepared via crystallization from hot supersaturated solutions. On the other hand, slow crystallization from RE lactate solutions at room temperature (from several weeks up to several months) yields crystalline precipitates of **2-Ln**, which contain X-ray quality single crystals. The Nd compound is an exception to this rule, as it always crystallizes in the **2-Ln** type. Presumably, this anomaly arises from the crystallographic instability of the **1-Ln** structure in the case of smaller Nd^3+^ ions.

A different situation was observed for heavy RE lactates. These compounds can crystallize in the form of hydrates [Ln(H_2_O)_2_Lact_3_]∙H_2_O (**4-Ln**), which are, however, unstable and readily eliminate non-coordinated water molecules upon drying (this behavior is discussed further in Section 2.3). Thus, **4-Ln** types were prepared upon the slow evaporation of the mother liquors at room temperature and stored in air to avoid water elimination. On the other hand, fast evaporation of RE lactate solutions with the subsequent drying of solid products in a desiccator yielded pure **3-Ln** (Ln = Sm–Lu, Y) in all cases. X-ray quality single crystals of both **3-Ln** and **4-Ln** can be obtained via crystallization from the respective mother liquors.

In addition to the differences in their chemical composition, RE lactate complexes of the **1-Ln**–**4-Ln** types exhibit major differences in their molecular and crystal structures, as discussed in the next section. It is worth noting that the starting reagent (HLact) was an enantiopure L-isomer. Therefore, all syntheses yielded chiral complexes, which in turn formed chiral crystals **1-Ln**–**4-Ln** with no inversion centers or mirror/glide planes present.

### 2.2. Crystal Structures of RE Lactates

Single-crystal XRD revealed the existence of four structural types among RE lactate complexes: 1D-polymeric [Ln(H_2_O)_2_Lact_3_]_∞_ (**1-Ln**), dimeric [Ln_2_(H_2_O)_5_Lact_6_]∙H_2_O (**2-Ln**), and monomeric ([Ln(H_2_O)_2_Lact_3_] (**3-Ln**) and [Ln(H_2_O)_2_Lact_3_]∙H_2_O (**4-Ln**)). Full details on the unit cell parameters and Ln–O bond lengths for all crystal structures determined are summarized in Tables S1–S3. For crystal structures refined from PXRD data, the corresponding PXRD patterns with Rietveld fits are provided in Figures S5 and S6. Data on the Continuous Shape Measures (CShM [55]) analysis of RE coordination polyhedra are summarized in Table S4.

#### 2.2.1. Crystal Structures of Type **1-Ln** (Ln = La, Pr)

**Figure 3:**
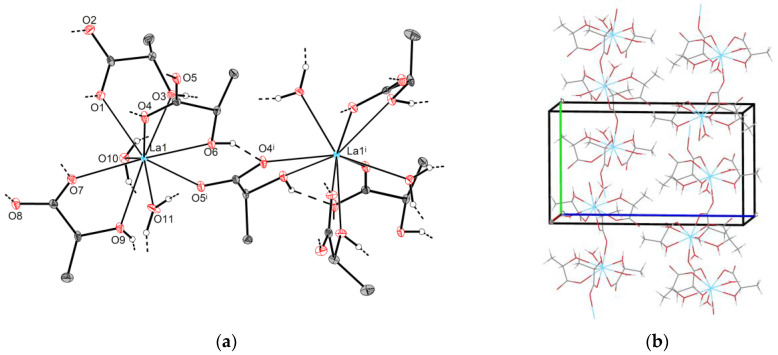
Crystal structure of **1-La**: (**a**) the selected fragment (thermal ellipsoids are depicted at the 50% probability level, C–H hydrogen atoms are omitted for clarity); (**b**) polymeric chains [La(H_2_O)_2_Lact_3_]_∞_. Symmetry code: (i) 1 − x, −0.5 + y, 1.5 − z.

As revealed from single-crystal XRD data, **1-La** exhibits a 1D-polymeric structure. **1-La** crystallizes in an orthorhombic lattice (space group *P2_1_2_1_2_1_*). The asymmetric part of the unit cell contains one lanthanum atom (La1), three Lact-anions, and two water molecules. La1 coordinates carboxyl and hydroxyl oxygen atoms from two chelating (O1∙∙∙O3, O7∙∙∙O9) and one chelating-bridging (O4∙∙∙O6) lactate ligands, two water molecules (O10, O11), and additionally one carboxyl oxygen atom of a chelating-bridging lactate anion (O5^i^) from the symmetry-related part (Figure 3a); thus, the CN of La1 is 9, and the coordination polyhedron is best described as a spherical tricapped trigonal prism (TCTPR-9, Table S4). It is worth noting that Ln–O bond lengths depend on the type of ligand atoms, being longer for α-hydroxyl groups (O3, O6, O9) and shorter for water molecules (O10, O11), chelating carboxyl atoms (O1, O4, O7), and bridging carboxyl atoms (O5^i^) in descending order (Table S2).

Adjacent lanthanum atoms are interlinked through chelating-bridging lactate ligands (O4∙∙∙O5∙∙∙O6) to form corrugated [La(H_2_O)_2_Lact_3_]_∞_ chains propagating along the [010] direction (Figure 3b). Neighboring [La(H_2_O)_2_Lact_3_]_∞_ chains are packed in a “herringbone” manner and are translated one into another by *2_1_*(*x*) and *2_1_*(*z*) screw axes as well as by crystallographic translations. Multiple intra- and interchain hydrogen bonds (d(O∙∙∙O)~2.64–2.85 Å) are present in the crystal. **1-Pr** possesses the same structural motif except for minor differences in the Ln–O coordination bond lengths, which are on average ~0.04 Å shorter than those of **1-La** (Table S2).

The main feature of the **1-Ln** structural type is the chelating-bridging coordination mode observed for some of the lactate ligands. Specifically, the latter not only form chelate cycles, they interconnect adjacent RE centers through an additional carboxyl oxygen atom. Analogous coordination behavior of lactate ligands was reported for the polymeric {[Ln(H_2_O)_2_Lact_2_](ClO_4_)}_∞_ (Ln = Eu, Tb) complexes [42,43].

#### 2.2.2. Crystal Structures of Type **2-Ln** (Ln = La, Ce, Pr)

Unlike the polymeric **1-Ln** compounds, crystal structures of [Ln_2_(H_2_O)_5_Lact_6_]∙H_2_O (**2-Ln**; Ln = La, Ce, Pr) consist of discrete dimeric molecules. All compounds in the **2-Ln** family exhibit triclinic symmetry (space group *P1*). The asymmetric part of the unit cell contains two Ln atoms (Ln1 an Ln2), six lactate ligands, and six water molecules. Ln1 coordinates carboxyl and hydroxyl oxygen atoms from three Lact-anions (O1∙∙∙O3, O4∙∙∙O6, O7∙∙∙O9) in a chelating mode. Additionally, Ln1 attaches two water molecules (O19, O20) and one carboxyl oxygen atom (O10) from a chelating-bridging lactate ion; therefore, the CN of Ln1 is 9, and the best fitting reference polyhedron is a spherical tricapped trigonal prism (TCTPR-9, Table S4). Ln–O bond lengths exhibit the same dependence on the type of ligand atoms as observed for **1-Ln** (Table S2). Similarly, Ln2 exhibits a nine-fold coordination environment; however, the arrangement of the ligands is different from that of Ln1. Specifically, Ln2 coordinates carboxyl and hydroxyl oxygen atoms from two chelating (O13∙∙∙O15, O16∙∙∙O18) and one chelating-bridging (O11∙∙∙O12) lactate anions, along with an additional three water molecules (O21, O22, O23). In contrast to the coordination environment of Ln1, Ln2 exhibits the longest Ln–O bonds for water ligands rather than α-hydroxyl groups (Table S2). The coordination polyhedron of Ln2 is best described as a “muffin” (MFF-9, Table S4).

**Figure 4:**
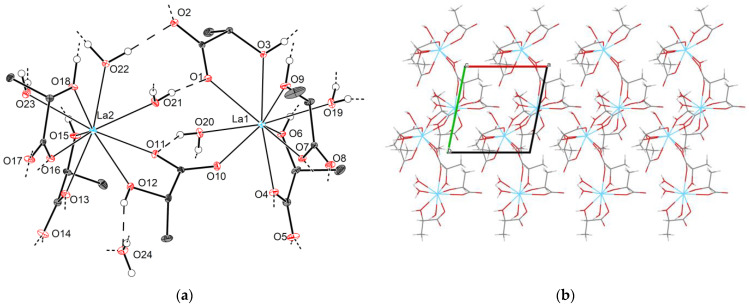
Crystal structure of **2-La**: (**a**) the asymmetric unit (one [La_2_(H_2_O)_5_Lact_6_] dimer and one non-coordinated water molecule); thermal ellipsoids are depicted at the 50% probability level, C–H hydrogen atoms are omitted for clarity, and “broken-off” dashed lines correspond to the intermolecular hydrogen bonds with adjacent structural units. (**b**) Crystal packing (view along the [001] direction).

As described for **1-Ln**, some of the lactate ions not only chelate RE ions through carboxyl and hydroxyl oxygen atoms, they bridge them through another carboxyl oxygen atom. Thus, Ln1–Ln2 pairs are linked with each other through chelating-bridging (O10∙∙∙O11∙∙∙O12) lactate ligands to form non-centrosymmetric dimers [Ln_2_(H_2_O)_5_Lact_6_] that are additionally stabilized by intramolecular hydrogen bonds (d(O∙∙∙O)~2.75–2.87 Å for **2-La**, Figure 4a). Additionally, neighboring [Ln_2_(H_2_O)_5_Lact_6_] species form multiple O–H∙∙∙O contacts with each other as well as with non-coordinated water molecules, yielding a three-dimensional hydrogen-bonded framework (Figure 4b).

#### 2.2.3. Crystal Structures of Types **3-Ln** and **4-Ln** (Ln = Sm–Lu, Y)

The decrease of the Ln^3+^ ionic radius in the lanthanide series reduces the preferable CN of the metal center and favors the formation of monomeric lactate complexes beginning from Sm^3+^. Thus, Ln lactates (Ln = Sm–Lu, Y) of both **3-Ln** and **4-Ln** types possess mononuclear structural motifs with virtually identical configurations of the Ln coordination environment while exhibiting differences at the supramolecular level.

**Figure 5:**
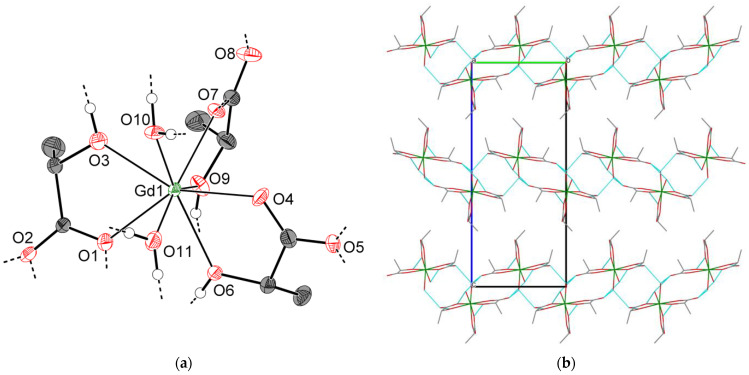
Crystal structure of **3-Gd**: (**a**) asymmetric unit (thermal ellipsoids are depicted at the 50% probability level, C–H hydrogen atoms are omitted for clarity, “broken-off” dashed lines correspond to the intermolecular hydrogen bonds with adjacent structural units); (**b**) projection of hydrogen-bonded layers (view along the [100] direction; dashed blue lines correspond to the O–H∙∙∙O hydrogen bonds; all hydrogen atoms are omitted for clarity).

All **3-Ln** compounds crystallize with orthorhombic unit cells (space group *P2_1_2_1_2_1_*). The asymmetric part of the unit cell contains one [Ln(H_2_O)Lact_3_] molecule. Ln1 coordinates carboxyl and hydroxyl oxygen atoms from three chelating lactate ligands (O1∙∙∙O3, O4∙∙∙O6, O7∙∙∙O9) and additional oxygen atoms from two water molecules (O10, O11) to form an eight-vertex coordination polyhedron (Figure 5a). The latter is well described by multiple reference shapes; the best fitting polyhedron with the minimal CShM parameter varies among the **3-Ln** group, being either a triangular dodecahedron TDD-8 or a square antiprism SAPR-8 (Table S4).

A detailed analysis of the crystal packing for **3-Ln** reveals the presence of hydrogen-bonded layers; their structure can be represented as follows. First, neighboring [Ln(H_2_O)Lact_3_] molecules form intermolecular ROH∙∙∙OOCR and HOH∙∙∙OOCR hydrogen bonds (d(O∙∙∙O)~2.62–2.71 Å for **3-Gd**) to yield infinite chain-like species [Ln(H_2_O)Lact_3_]_∞_ propagating along the [100] direction, with the shortest Ln∙∙∙Ln separation being 5.7542(2) Å for **3-Gd**. In turn, [Ln(H_2_O)Lact_3_]_∞_ species link with each other through additional HOH∙∙∙OOCR hydrogen bonds (d(O∙∙∙O)~2.67–2.80 Å for **3-Gd**) into extended corrugated layers parallel to the (001) plane (Figure 5b), which interact primarily via weak van der Waals forces.

It is worth noting that the monoclinic crystal structure of [Y(H_2_O)_2_Lact_3_] (henceforth **5-Y**) reported by Yapryntsev et al. [44] exhibits a similar structural motif that differs from **3-Ln** in the crystallographic symmetry and arrangement of hydrogen-bonded species. To be more precise, the crystal of **5-Y** consists of mononuclear [Y(H_2_O)_2_Lact_3_] molecules interlinked by hydrogen bonds into chain-like species [Y(H_2_O)_2_Lact_3_]_∞_, which in turn form corrugated layers. Despite possessing a virtually identical topology of an isolated hydrogen-bonded layer, **3-Ln** and **5-Y** differ in the packing of those. In the former case, neighboring layers are translated one into another through *2_1_*(*y*) and *2_1_*(*z*) screw axes and adjacent layers are rotated by 180° along the [001] direction relative to each other. Therefore, the layer orientations alternate, and an ABAB packing motif is observed (Figure S7a). In the case of **5-Y**, adjacent layers are simply transformed into each other through the ***c*** crystallographic translation, which has a small in-layer shift because the ***β*** angle slightly deviates from 90°. In other words, the arrangement of the layers in **5-Y** can be described as a distorted AAA motif (Figure S7b). Thus, **3-Ln** and **5-Y** are polytypes. Variable layer arrangements (i.e., polytypism) for RE lactates are possible due to the weak non-specific interlayer interactions, as all hydrogen bonds are intralayer. A similar case of polytypism was previously reported for yttrium and heavy lanthanide (Ho–Lu) propionates [18].

**Figure 6:**
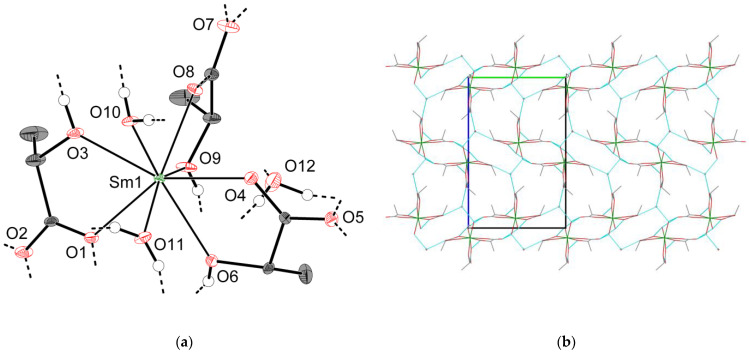
Crystal structure of **4-Sm**: (**a**) asymmetric unit (thermal ellipsoids are depicted at the 50% probability level, C–H hydrogen atoms are omitted for clarity, “broken-off” dashed lines correspond to the intermolecular hydrogen bonds with adjacent structural units); (**b**) projection of the hydrogen-bonded framework (view along the [100] direction; dashed blue lines correspond to the O–H∙∙∙O hydrogen bonds; isolated spheres in the packing cavities correspond to the non-coordinated water molecules; all hydrogen atoms are omitted for clarity).

As described for **3-Ln**, the crystal structures of **4-Ln** consist of discrete [Ln(H_2_O)_2_Lact_3_] molecules (Figure 6a) interlinked into hydrogen-bonded species [Ln(H_2_O)_2_Lact_3_]_∞_ (d(O∙∙∙O)~2.66–2.77 Å, shortest d(Ln∙∙∙Ln) 5.8257(5) Å for **4-Sm**), and the overall arrangement of molecules in the unit cell is very similar to that of **3-Ln**. However, unlike **3-Ln**, neighboring [Ln(H_2_O)_2_Lact_3_]_∞_ species in **4-Ln** are linked through hydrogen bonds (d(O∙∙∙O)~2.70 Å for **4-Sm**) into double chains {[Ln(H_2_O)_2_Lact_3_]_∞_}_2_ rather than extended layers. The *b* unit cell parameter, which is primarily responsible for the interchain Ln∙∙∙Ln separation, turns out to be significantly elongated with respect to that of **3-Ln** (13.3765(12) and 10.7147(3) Å for **4-Sm** and **3-Gd**, respectively). Packing cavities between {[Ln(H_2_O)_2_Lact_3_]_∞_}_2_ double chains are occupied by non-coordinated water molecules, which form three hydrogen bonds each with adjacent {[Ln(H_2_O)_2_Lact_3_]_∞_}_2_ chains (d(O∙∙∙O)~2.69–2.85 Å for **4-Sm**) to yield a hydrogen-bonded 3D-framework (Figure 6b).

As mentioned in Section 2.1, RE carboxylates are structurally flexible, and addition/elimination of solvent molecules often leads to the drastic changes in the molecular structure or/and crystal packing [56]. Thus, compounds **3-Ln** and **4-Ln** represent a peculiar example of RE carboxylates where the introduction of ancillary water molecules retains the coordination environment of Ln centers as well as certain features at the supramolecular level (i.e., [Ln(H_2_O)_2_Lact_3_]_∞_ chain-like species).

To summarize the data on RE lactate structures, a number of conclusions should be pointed out. First of all, Lact-anions are prone to forming stable five-membered chelate rings with RE cations through COO- and OH-groups rather than the four-membered chelate rings abundant among non-substituted RE carboxylates [13,18,56,57]. In the case of medium- and small-radius Ln^3+^ cations (Sm–Lu, Y), formation of three chelating cycles along with coordination of two water molecules is enough to fulfill the coordination preferences of the metal center without any ancillary chemical interactions. Furthermore, lactate ligands can form additional COO–Ln bonds (i.e., exhibit a chelating-bridging mode) if the coordination sphere of Ln centers cannot be fully saturated by chelate cycles. This occurs, for instance, for larger RE (La, Ce–Nd) lactates where the nine-fold coordination polyhedron of Ln is completed through the additional Ln–O bonding with neighboring structural units, yielding polymeric (**1-Ln**) or binuclear (**2-Ln**) structures depending on the amount of coordinated water molecules. Formation of coordination polymers is possible for medium-radius Eu^3+^ and Tb^3+^ cations if lactate ligands are partially substituted with non-coordinating anions (e.g., in the structures of {[Ln(H_2_O)_2_Lact_2_](ClO_4_)}_∞_ [42,43]). In this case, two chelating cycles cannot provide the desired eight-fold coordination environment of RE cations; consequently, the coordination mode for all lactate ligands switches to chelating-bridging to yield a 2D-polymeric network.

### 2.3. Thermal Decomposition of RE Lactates

**Figure 7:**
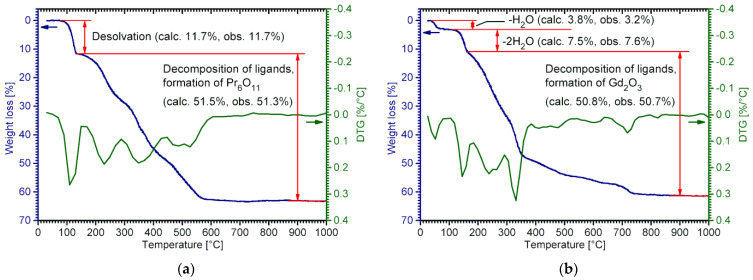
TG-DTG curves recorded for: (**a**) **2-Pr** and (**b**) **4-Gd**. A full set of TG-DTG curves for RE lactates is shown in Figures S1–S4.

According to the TGA data, all RE lactates undergo multi-step thermal decomposition. The first stage corresponds to the desolvation, and its characteristic temperature significantly differs for the **1-Ln**–**4-Ln** structural types. In general, compounds with non-coordinated water molecules (**2-Ln** and **4-Ln**) undergo desolvation at lower temperatures than those with only coordinated water molecules (**1-Ln**, **3-Ln**). Indeed, [Ln(H_2_O)_2_Lact_3_]_∞_ (**1-Ln**; Ln = La, Ce, Pr) begin to decompose at ca. 110–120 °C (Figure S1). An analogous process for [Ln(H_2_O)_2_Lact_3_] (**3-Ln**; Ln = Sm–Lu, Y) generally starts at 100–120 °C (Figures S2 and S3). On the other hand, hydrated complexes [Ln_2_(H_2_O)_5_Lact_6_]∙H_2_O (**2-Ln**; Ln = La, Ce–Nd) decompose at temperatures higher than ~80 °C; furthermore, both coordinated and non-coordinated water molecules are removed in a single stage with no intermediate products, e.g., **1-Ln**, being formed (Figure 7a and Figure S1).

For the hydrated compounds [Ln(H_2_O)_2_Lact_3_]∙H_2_O (**4-Ln**; Ln = Sm–Gd, Y), TG-curves reveal a two-step desolvation process. Namely, these partially decompose upon minor heating (30–60 °C) to form the respective [Ln(H_2_O)_2_Lact_3_] (**3-Ln**) complexes, which remain stable up to 100–120 °C and then undergo complete desolvation (Figure 7b and Figure S4).

Upon further heating, the products of desolvation (anhydrous RE lactates) decompose through multiple overlapping stages that presumably refer to the competing processes of the pyrolysis and combustion of organic ligands. As follows from the TGA data, the thermal stability of anhydrous RE lactates slightly decreases in the Ln series, with a pronounced anomaly in the case of Ce lactate. The latter decomposes at significantly lower temperatures (above ~125 °C, as observed for **2-Ce**—Figure S1) than the neighboring La and Pr compounds, presumably due to the oxidation of Ce^III^. The final step on TG-curves (except for **1-Ce** and **2-Ce**, which immediately yield CeO_2_ after the decomposition of ligands) corresponds to the decomposition of RE carbonates or/and oxocarbonates.

**Figure 8:**
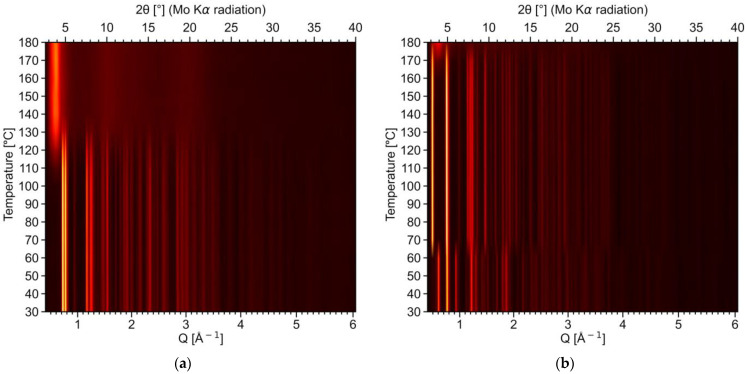
VT-PXRD data (Mo Kα radiation) in the form of color maps for: (**a**) **2-Pr** and (**b**) **4-Gd**. *Q* refers to the modulus of a scattering vector transfer: *Q =* 4*π∙sinθ/λ*. Sections of the color maps at selected temperatures are shown in Figure S8.

The differences in the desolvation processes for **2-Ln** and **4-Ln** have additionally been proven by means of variable-temperature PXRD (VT-PXRD) using the example of the **2-Pr** and **4-Gd** compounds (Figure 8 and Figure S8). In the former case, the PXRD pattern remains virtually unchanged up to 100 °C, i.e., the native [Pr_2_(H_2_O)_5_Lact_6_]∙H_2_O (**2-Pr**) phase remains stable in this temperature region (Figure 8a). Upon further heating, the initial hydrated complex undergoes gradual decomposition, eventually yielding an amorphous product (presumably, anhydrous Pr lactate) at 140 °C that exhibits only a diffuse X-ray scattering halo rather than sharp X-ray diffraction peaks. It is worth noting that no intermediate phases (e.g., **1-Pr**—[Pr(H_2_O)_2_Lact_3_]_∞_) were observed during the desolvation process; therefore, **2-Pr** indeed eliminates all water molecules in a single stage.

A different behavior was observed for **4-Gd**. As follows from the VT-PXRD data, the initial [Gd(H_2_O)_2_Lact_3_]∙H_2_O phase retains up to 60 °C. At higher temperatures the PXRD pattern changes rapidly, indicating the formation of the pure **3-Gd** phase upon the removal of non-coordinated water molecules (Figure 8b and Figure S8). In other words, the desolvation of [Gd(H_2_O)_2_Lact_3_**]**∙H_2_O (**4-Gd**) is actually a two-step process involving an intermediate [Gd(H_2_O)_2_Lact_3_] (**3-Gd**) product, as observed in the TGA experiments (Figure 7b). The product of total desolvation, which is formed above 160–170 °C, is amorphous.

The observed differences in the thermal behavior of RE lactate hydrates can be explained on the basis of their crystal structures. Indeed, the transition from **4-Ln** to **3-Ln** only involves the removal of non-coordinated water molecules and slight lattice distortions. On the other hand, **2-Ln** and **1-Ln** exhibit major structural differences, and the former cannot be simply transformed into the latter through a solid-state process at relatively low (*ca.* 100 °C) temperatures.

### 2.4. Ln_2_O_3_ Thin Films

Thin films of RE sesquioxides have been previously used as planarization sublayers for second-generation HTS tapes [58,59,60]; the major characteristic of such films is their root-mean-square roughness (henceforth S_q_), which is expected to be lower than that of the original substrate. Highly smooth coatings are usually fabricated from amorphous or nanocrystalline oxides, which are formed upon the low-temperature (500–600 °C) heat treatment of the respective metal–organic precursors [60].

**Figure 9:**
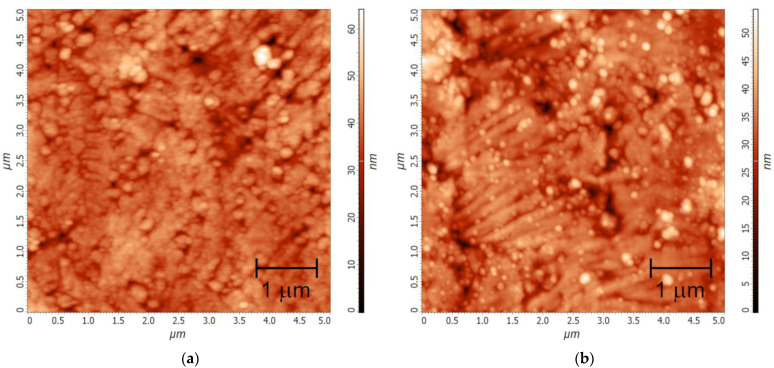
AFM topography (5 × 5 μm^2^ scans) of Ln_2_O_3_/HC276 thin films: (**a**) **Y-F1** and (**b**) **La-F1**.

A Y_2_O_3_ thin film (**Y-F1**) was deposited onto an as-rolled hastelloy HC276 tape with a native surface possessing an S_q_ value of 8.2 nm (Figure S9a). As estimated from AFM data, the **Y-F1** coating has a significantly enhanced smoothness (S_q_ = 4.9 nm for a 5 × 5 μm^2^ scan area, Figure 9a), i.e., a planarization effect is achieved even through only one deposition cycle. The θ–θ XRD scan of **Y-F1** reveals no Bragg reflections except those of the substrate material (Figure S10); therefore, the **Y-F1** film exhibits no crystallinity, similarly to the Y_2_O_3_ film obtained from the yttrium acetate precursor [60]. One-cycle deposition of a La_2_O_3_ film onto an as-rolled HC276 substrate (**La-F1**) reduces its mean roughness, yielding an S_q_ value of 5.1 nm (5 × 5 μm^2^ scan area; Figure 9b), which is close to that of the **Y-F1** coating. In other words, both **Y-F1** and **La-F1** make a comparable planarization effect. The smoothness of the surface can be further enhanced via multiple cycles of deposition that makes it possible to produce planar buffer layers for the fabrication of second-generation HTS tapes. To highlight this effect, preliminarily electropolished HC276 tape with a residual S_q_ of 1.1 nm (Figure S9b) was coated with a La-based thin film (**La-F2**) deposited from a twice-diluted solution of lanthanum lactate-based precursor (**La-S2**) through seven cycles of deposition. As a result, a highly smooth (S_q_ = 0.45 nm, Figure S11) surface was obtained.

## 3. Materials and Methods

Ln(NO_3_)_3_∙nH_2_O (“Reakhim, Moscow, Russia”, reagent grade), Ln_2_(CO_3_)_3_∙mH_2_O (“Reakhim”, reagent grade), aqueous ammonia (C_NH3_ = 16 mol/l, “Reakhim”, reagent grade), aqueous lactic acid (HLact, 80%wt, “Reakhim”, analytical grade), isopropanol (^i^PrOH, “NPP ‘Gamma’, Moscow, Russia”, reagent grade), and diethylenetriamine (DETA, “Sigma-Aldrich, Darmstadt, Germany”, 99%) were purchased from local suppliers and used without further purification. Polycrystalline hastelloy HC276 tapes were purchased from SuperOx LLC (Moscow, Russia).

TG-DTA data (air atmosphere, temperature range 25–1000 °C, heating rate 10 °C/min, sample mass ca. 50 mg) were recorded on a Derivatograph Q-1500 D instrument (MOM, Budapest,). Measurements of C and H contents were carried out using an Elementar Vario MICRO cube instrument (Elementar Analysensysteme GmbH, Langenselbold, Germany). Ln content was determined by complexometric titration (acetate buffer, Xylenol orange) as well as from the TGA data at 1000 °C.

FTIR spectra were recorded on a Perkin-Elmer Spectrum One FTIR spectrometer (PerkinElmer Inc., Waltham, USA) in the attenuated total reflectance (ATR) geometry in the wavenumber range of 650−4000 cm^−1^.

PXRD data of powder samples at 300 K were collected using a Rigaku MINIFLEX (600 W X-ray tube, Cu Kα radiation, Kβ-filter, symmetrical reflection θ−2θ mode, D/teX Ultra 1D-linear detector, Rigaku, Tokyo, Japan) laboratory diffractometer.

Variable-temperature PXRD (VT-PXRD) experiments were performed in a temperature range of 30–180 °C in the Debye–Scherrer geometry (2θ range 2.6–41.2°) on a Bruker D8 QUEST diffractometer (Photon III CMOS area detector, Mo Kα radiation, Montel optics, Bruker AXS Inc., Madison, USA) equipped with a hot air blower. Samples were placed into opened Kapton^®^ capillaries of 0.5 mm diameter. The in situ heating experiment was performed in a stepwise heating mode (10 °C/step) with an average heating rate of 3 °C/min. As described recently, 2D diffraction patterns were processed using FormagiX v.0.9.5. software and calibrated with an NIST SRM660c LaB_6_ reference sample [61].

The phase composition of thin films was studied by means of XRD using a Rigaku SmartLab diffractometer (Cu Kα_1_ radiation, incident beam Ge (220)×2 monochromator, symmetrical reflection θ–θ mode). The surface morphology and roughness of films were examined by atomic force microscopy (AFM) on an NT-MDT NTegra Aura instrument (NT-MDT, Moscow, Russia) in semi-contact mode.

### 3.1. Synthesis and Crystal Growth of RE Lactates

RE lactate complexes LnLact_3_∙nH_2_O (Ln = Y, La, Ce–Nd, Sm–Lu; *n* = 2–3) of four structural types (**1-Ln**–**4-Ln**) were obtained from aqueous solutions by three different synthetic routes, as described below. The chemical and phase composition of all obtained products were confirmed by means of CHN, TG-DTA, FTIR, and XRD analyses.

**Procedure I.** A 10 mmol amount of Ln(NO_3_)_3_∙6H_2_O was dissolved in 40 mL of distilled water. After that, 2.2 mL of aqueous ammonia (C_NH3_ = 16 M, ca. 35 mmol NH_3_) was added upon stirring. The resulting precipitate was centrifuged and washed with distilled water three times, then suspended in 10 mL of H_2_O; next, 30 mmol of HLact was added to the reaction mixture. The latter was stirred at elevated temperatures (ca. 75 °C) to obtain a clear solution. The resulting solution for Ln = La–Nd was slowly cooled to room temperature and stored in air for evaporation and crystallization within 3–8 weeks, which yielded crystals of **2-Ln**. Alternatively, the same solution was evaporated upon heating until the crystallization began (to ca. 50% of its initial volume) and then cooled to room temperature. Precipitates of the **1-Ln** phase for Ln = La–Pr, as well as **3-Ln** or **4-Ln** for Ln = Sm–Lu and Y, were obtained in this manner.

The resulting precipitate was filtered off, washed with several milliliters of H_2_O and EtOH, and stored in a desiccator over NaOH (**1-Ln**, **2-Ln**, and **3-Ln**) or in air (**4-Ln**). The yields were 65–85%.

**Procedure II.** A 5 mmol amount of Ln_2_(CO_3_)_3_∙nH_2_O was dispersed in 60 mL of water and 40 mmol of HLact was added to the reaction mixture. The latter was refluxed at 100 °C for 2 h and cooled to the room temperature, which yielded a fine crystalline precipitate. The latter was treated in the same manner as described above. The yield was ca. 85%.

**Procedure III.** A 10 mmol amount of Ln(NO_3_)_3_∙6H_2_O was dissolved in 40 mL of distilled water. An aqueous solution of NH_4_Lact was prepared separately by mixing 1.9 mL of aqueous ammonia (C_NH3_ = 16 M, ca. 30 mmol NH_3_) and 31 mmol of HLact in 40 mL of water. The prepared solutions were mixed upon vigorous stirring and evaporated to ca. 50% of the initial volume. Crystallization occurred within 0.5–24 h after the synthesis, and the resulting precipitate was treated in the same manner as described above. The yield was ca. 60%.

In all procedures, the mother liquors left after filtration were slowly evaporated in ambient conditions for several days; X-ray quality single crystals of **3-Gd** and **4-Sm** were obtained in this manner. Crystals of **1-La** and **1-Pr** were grown from hot supersaturated solutions of the respective RE lactates. Single crystals of **2-La**, **2-Ce**, and **2-Pr** were harvested directly from the respective bulk products prepared via slow (3–8 weeks) crystallization in ambient conditions.

*[La(H_2_O)_2_Lact_3_]_∞_* (**1-La**). Calc. for C_9_H_19_LaO_11_ (%): La, 31.42; C, 24.45; H, 4.33. Found (%): La, 31.3; C, 24.8; H, 4.5. FTIR (ATR, ν, cm^−1^): 3338sh, 3264, 2992w, 2935vw, 2876vw (νOH, νCH); 1705w; 1698w; 1668w (δH_2_O); 1590sh, 1557s (ν_as_COO); 1486sh, 1477sh, 1465 (δ_as_CH_3_); 1439vw; 1425w, 1391 (ν_s_COO, δCOH); 1375w; 1365, 1359sh, 1351sh (δ_s_CH_3_); 1319; 1284; 1242s; 1130s; 1117s; 1093; 1055; 1037; 937; 864; 815; 773, 765 (δCOO); 660.
*[Ce(H_2_O)_2_Lact_3_]_∞_* (**1-Ce**). Calc. for C_9_H_19_CeO_11_ (%): Ce, 31.60; C, 24.38; H, 4.32. Found (%): Ce, 31.5; C, 24.3; H, 4.3. FTIR (ATR, ν, cm^−1^): 3335sh, 3256, 2993w, 2936vw, 2877vw (νOH, νCH); 1705w; 1695sh; 1664w (δH_2_O); 1588sh, 1557s (ν_as_COO); 1485sh, 1478sh, 1464 (δ_as_CH_3_); 1439w; 1425w, 1388 (ν_s_COO, δCOH); 1364, 1359, 1351 (δ_s_CH_3_); 1318; 1283; 1243s; 1126s; 1117s; 1093; 1055; 1036; 937; 864; 815; 774, 764 (δCOO); 660.
*[Pr(H_2_O)_2_Lact_3_]_∞_* (**1-Pr**). Calc. for C_9_H_19_O_11_Pr (%): Pr, 31.73. Found (%): Pr, 31.6. FTIR (ATR, ν, cm^−1^): 3338sh, 3255, 2995w, 2939vw, 2879vw (νOH, νCH); 1704vw; 1694vw; 1662w (δH_2_O); 1591sh, 1558 (ν_as_COO); 1480, 1466 (δ_as_CH_3_); 1440w; 1426w, 1391 (ν_s_COO, δCOH); 1375w; 1365, 1358, 1352sh (δ_s_CH_3_); 1330; 1319; 1284; 1245s; 1130s; 1118s; 1094; 1056; 1037; 938; 865; 817; 774, 764 (δCOO); 660.
*[La_2_(H_2_O)_5_Lact_6_]∙H_2_O* (**2-La**). Calc. for C_18_H_42_La_2_O_24_ (%): La, 30.19; C, 23.49; H, 4.60. Found (%): La, 30.4; C, 23.6; H, 4.5. FTIR (ATR, ν, cm^−1^): 3562sh, 3303sh, 3170, 2985vw, 2941vw (νOH, νCH); 1665w, 1653sh, 1645sh (δH_2_O); 1568s (ν_as_COO); 1475 (δ_as_CH_3_); 1424sh, 1395 (ν_s_COO, δCOH); 1378sh; 1363 (δ_s_CH_3_); 1320, 1277sh, 1268; 1232w; 1127; 1116s; 1089; 1041s; 932; 865; 845w; 774 (δCOO); 663.
*[Ce_2_(H_2_O)_5_Lact_6_]∙H_2_O* (**2-Ce**). Calc. for C_18_H_42_Ce_2_O_24_ (%): Ce, 30.37; C, 23.43; H, 4.59. Found (%): Ce, 31.2; C, 23.0; H, 4.6. FTIR (ATR, ν, cm^−1^): 3319sh, 3203, 2986w, 2941vw (νOH, νCH); 1662vw, 1645sh (δH_2_O); 1564s (ν_as_COO); 1471vw, 1465sh, 1456sh (δ_as_CH_3_); 1423sh, 1395 (ν_s_COO, δCOH); 1387sh; 1377sh; 1362 (δ_s_CH_3_); 1319; 1280sh; 1266w; 1230sh; 1124sh; 1116s; 1089; 1041s; 932; 865; 845w; 819w; 774 (δCOO); 707sh; 660.
*[Pr_2_(H_2_O)_5_Lact_6_]∙H_2_O* (**2-Pr**). Calc. for C_18_H_42_O_24_Pr_2_ (%): Pr, 30.49. Found (%): Pr, 30.6. FTIR (ATR, ν, cm^−1^): 3569vw, 3328sh, 3199, 2985vw, 2941vw (νOH, νCH); 1660, 1650sh (δH_2_O); 1567s, 1556sh (ν_as_COO); 1475 (δ_as_CH_3_); 1426sh, 1398 (ν_s_COO, δCOH); 1389sh; 1384sh; 1376sh; 1363 (δ_s_CH_3_); 1320; 1306sh; 1279w; 1266; 1126; 1116s; 1090; 1042s; 933w; 866; 848w; 816wv; 774 (δCOO); 711sh; 663.
*[Nd_2_(H_2_O)_5_Lact_6_]∙H_2_O* (**2-Nd**). Calc. for C_18_H_42_Nd_2_O_24_ (%): Nd, 30.99. Found (%): Nd, 32.0. FTIR (ATR, ν, cm^−1^): 3319sh, 3195, 2980sh, 2975sh, 2967sh, 2942vw, 2936sh, 2897w, 2880w (νOH, νCH); 1659w, 1651sh (δH_2_O); 1570s, 1559sh (ν_as_COO); 1471, 1450vw (δ_as_CH_3_); 1423sh, 1399 (ν_s_COO, δCOH); 1385sh; 1363 (δ_s_CH_3_); 1320; 1280; 1266; 1223; 1126; 1116s; 1092; 1042s; 933; 866; 849; 812sh; 775 (δCOO); 710sh; 672; 663.
*[Sm(H_2_O)_2_Lact_3_]* (**3-Sm**). Calc. for C_9_H_19_O_11_Sm (%): Sm, 33.15; C, 23.83; H, 4.22. Found (%): Sm, 33.2; C, 23.6; H, 4.3. FTIR (ATR, ν, cm^−1^): 3399, 3159sh, 3054, 2997, 2989, 2980sh, 2957w, 2943sh, 2883vw (νOH, νCH); 1668sh (δH_2_O); 1577s (ν_as_COO); 1481, 1470, 1456sh (δ_as_CH_3_); 1427sh, 1407sh, 1404sh, 1391s (ν_s_COO, δCOH); 1365, 1358 (δ_s_CH_3_); 1320sh; 1314s; 1281; 1270sh; 1263; 1111s; 1094; 1045s; 932; 863; 782, 776sh (δCOO); 759; 709; 665sh.
*[Eu(H_2_O)_2_Lact_3_]* (**3-Eu**). Calc. for C_9_H_19_EuO_11_ (%): Eu, 33.38. Found (%): Eu, 33.7. FTIR (ATR, ν, cm^−1^): 3403, 3158sh, 3058, 2997, 2989, 2980sh, 2958w, 2938w, 2885sh (νOH, νCH); 1663sh (δH_2_O); 1583s (ν_as_COO); 1482, 1469, 1457sh (δ_as_CH_3_); 1427sh, 1408sh, 1393s (ν_s_COO, δCOH); 1365, 1359 (δ_s_CH_3_); 1321sh; 1314s; 1282; 1272; 1265; 1112s; 1095; 1046s; 932; 863; 783, 776sh (δCOO); 758; 712; 651.
*[Gd(H_2_O)_2_Lact_3_]* (**3-Gd**). Calc. for C_9_H_19_GdO_11_ (%): Gd, 34.15. Found (%): Gd, 34.1. FTIR (ATR, ν, cm^−1^): 3403, 3161sh, 3074, 3013vw, 2998w, 2978vw, 2960w, 2946w, 2845vw (νOH, νCH); 1668sh (δH_2_O); 1585s (ν_as_COO); 1480, 1465 (δ_as_CH_3_); 1409sh, 1394s (ν_s_COO, δCOH); 1372, 1358 (δ_s_CH_3_); 1314s; 1278sh; 1263; 1115s; 1092sh; 1045s; 933; 864; 787, 768, 753sh (δCOO); 710; 651.
*[Tb(H_2_O)_2_Lact_3_]* (**3-Tb**). Calc. for C_9_H_19_O_11_Tb (%): Tb, 34.39. Found (%): Tb, 34.1. FTIR (ATR, ν, cm^−1^): 3403, 3151sh, 3061, 2997, 2989, 2981sh, 2955w, 2938w, 2883sh (νOH, νCH); 1661sh (δH_2_O); 1580s (ν_as_COO); 1483, 1468, 1456sh (δ_as_CH_3_); 1428sh, 1409sh, 1393s (ν_s_COO, δCOH); 1365, 1359 (δ_s_CH_3_); 1321sh; 1315s; 1281sh; 1271sh; 1264; 1112s; 1096; 1046s; 933; 864; 783, 776sh (δCOO); 759sh; 715; 666sh; 651.
*[Dy(H_2_O)_2_Lact_3_]* (**3-Dy**). Calc. for C_9_H_19_DyO_11_ (%): Dy, 34.89. Found (%): Dy, 35.3. FTIR (ATR, ν, cm^−1^): 3405, 3161sh, 3061, 2997w, 2990w, 2959sh, 2941sh, 2881vw (νOH, νCH); 1581s (ν_as_COO); 1485, 1468 (δ_as_CH_3_); 1413sh, 1394s (ν_s_COO, δCOH); 1366w, 1359 (δ_s_CH_3_); 1315s; 1282sh; 1273sh; 1265; 1123sh; 1112s; 1096; 1047s; 934; 864; 784, 775sh, 760sh (δCOO); 716; 651.
*[Ho(H_2_O)_2_Lact_3_]* (**3-Ho**). Calc. for C_9_H_19_HoO_11_ (%): Ho, 35.23; C, 23.09; H, 4.09. Found (%): Ho, 35.9; C, 22.8; H, 4.2. FTIR (ATR, ν, cm^−1^): 3405, 3158sh, 3064, 2981, 2967, 2942sh, 2880vw (νOH, νCH); 1666sh (δH_2_O); 1583s (ν_as_COO); 1485, 1469 (δ_as_CH_3_); 1415sh, 1395s (ν_s_COO, δCOH); 1366, 1359 (δ_s_CH_3_); 1323sh; 1315; 1283sh; 1273sh; 1266; 1124sh; 1113s; 1096; 1047s; 934; 865; 785, 775sh (δCOO); 719; 651.
*[Er(H_2_O)_2_Lact_3_]* (**3-Er**). Calc. for C_9_H_19_ErO_11_ (%): Er, 35.55. Found (%): Er, 35.6. FTIR (ATR, ν, cm^−1^): 3406, 3157sh, 3059, 3019vw, 2997sh, 2990, 2981sh, 2961w, 2940vw, 2878sh (νOH, νCH); 1666sh (δH_2_O); 1584s (ν_as_COO); 1487, 1469 (δ_as_CH_3_); 1434sh, 1416sh, 1397s (ν_s_COO, δCOH); 1378sh; 1366, 1359 (δ_s_CH_3_); 1324sh; 1316; 1283; 1273sh; 1267; 1125w; 1113s; 1096; 1047s; 935; 865; 786, 776sh (δCOO); 722; 673vw; 654.
*[Tm(H_2_O)_2_Lact_3_]* (**3-Tm**). Calc. for C_9_H_19_O_11_Tm (%): Tm, 35.78. Found (%): Tm, 37.2. FTIR (ATR, ν, cm^−1^): 3408, 3158sh, 3062, 2996sh, 2989, 2983sh, 2966w, 2950vw 2931vw, 2879vw (νOH, νCH); 1663sh (δH_2_O); 1583s (ν_as_COO); 1487, 1469 (δ_as_CH_3_); 1434sh, 1413sh, 1396s (ν_s_COO, δCOH); 1380; 1366, 1359 (δ_s_CH_3_); 1322sh; 1316s; 1282sh; 1272sh; 1266; 1113s; 1097; 1048s; 935; 865; 785, 777sh (δCOO); 723.
*[Yb(H_2_O)_2_Lact_3_]* (**3-Yb**). Calc. for C_9_H_19_O_11_Yb (%): Yb, 36.33. Found (%): Yb, 35.6. FTIR (ATR, ν, cm^−1^): 3419, 3167sh, 3062, 3001, 2983, 2941, 2892w (νOH, νCH); 1663sh (δH_2_O); 1583s (ν_as_COO); 1487, 1471 (δ_as_CH_3_); 1434sh, 1414sh, 1398s (ν_s_COO, δCOH); 1379; 1364 (δ_s_CH_3_); 1318s; 1284; 1267; 1126; 1114s; 1102sh; 1098sh; 1048s; 935; 866; 786, 777sh (δCOO); 722; 651.
*[Lu(H_2_O)_2_Lact_3_]* (**3-Lu**). Calc. for C_9_H_19_LuO_11_ (%): Lu, 36.59. Found (%): Lu, 37.8. FTIR (ATR, ν, cm^−1^): 3408, 3146sh, 3066, 2996sh, 2990, 2983sh, 2941sh, 2879vw (νOH, νCH); 1667sh (δH_2_O); 1584s (ν_as_COO); 1485, 1470 (δ_as_CH_3_); 1434sh, 1414sh, 1399s (ν_s_COO, δCOH); 1380; 1366, 1360 (δ_s_CH_3_); 1323sh; 1316s; 1282sh, 1273sh, 1268; 1113s; 1098; 1049s; 936; 866; 786, 776sh (δCOO); 727.
*[Y(H_2_O)_2_Lact_3_]* (**3-Y**). Calc. for C_9_H_19_O_11_Y (%): Y, 22.67; C, 27.57; H, 4.88. Found (%): Y, 23.4; C, 27.3; H, 4.9. FTIR (ATR, ν, cm^−1^): 3407, 3159sh, 3062, 2996sh, 2990, 2980sh, 2941sh, 2881vw (ν_s_COO, δCOH); 1583 (ν_as_COO); 1484, 1469 (δ_as_CH_3_); 1416sh, 1396s (ν_s_COO, δCOH); 1377sh, 1365, 1359 (δ_s_CH_3_); 1323sh; 1315s; 1283sh; 1275sh; 1266; 1124sh; 1113s; 1096; 1047s; 934; 865; 785, 775sh (δCOO); 719; 652.
*[Sm(H_2_O)_2_Lact_3_]∙H_2_O* (**4-Sm**). Calc. for C_9_H_21_O_12_Sm (%): Sm, 31.88. Found (%): Sm, 31.8. FTIR (ATR, ν, cm^–1^): 3492vw, 3399w, 3157sh, 3068, 2997w, 2989w, 2979sh, 2959vw, 2941sh. 2883vw (νOH, νCH); 1634sh (δH_2_O); 1582s (ν_as_COO); 1480, 1467 (δ_as_CH_3_); 1407sh, 1391s (ν_s_COO, δCOH); 1367, 1358 (δ_s_CH_3_); 1322sh; 1314s; 1280sh; 1271sh; 1264; 1114s; 1094; 1045s; 932; 863; 782, 774sh (δCOO); 707; 650.
*[Eu(H_2_O)_2_Lact_3_]∙*H_2_O (**4-Eu**). Calc. for C_9_H_21_EuO_12_ (%): Eu, 32.11. Found (%): Eu, 32.2. FTIR (ATR, ν, cm^–1^): 3492vw, 3402vw, 3157sh, 3084, 3014w, 2999w, 2978w, 2960vw, 2949vw (νOH, νCH); 1636 (δH_2_O); 1588s (ν_as_COO); 1479, 1465 (δ_as_CH_3_); 1409s, 1396sh (ν_s_COO, δCOH); 1372, 1358 (δ_s_CH_3_); 1315s; 1278; 1264; 1116s; 1092sh; 1045s; 931; 864; 788, 768 (δCOO); 705.
*[Y(H_2_O)_2_Lact_3_]∙*H_2_O (**4-Y**). Calc. for C_9_H_21_O_12_Y (%): Y, 21.68. Found (%): Y, 21.5. FTIR (ATR, ν, cm^–1^): 3492vw, 3405sh, 3170sh, 3086, 3014w. 2999w, 2979w, 2963w, 2946w (νOH, νCH); 1639 (δH_2_O); 1591s (ν_as_COO); 1482sh, 1466 (δ_as_CH_3_); 1412, 1401 (ν_s_COO, δCOH); 1373, 1359 (δ_s_CH_3_); 1316s; 1279; 1265; 1115s; 1092w; 1051sh; 1045s; 934; 866; 790, 769 (δCOO); 708w; 677w.

### 3.2. Preparation of Precursor Solutions and Deposition of Ln_2_O_3_ Thin Films

Thin films of Y_2_O_3_ and La_2_O_3_ oxides were deposited onto metallic polycrystalline cold-rolled hastelloy HC276 substrates (length 100 mm, width 12 mm, thickness 110 μm) by the MOCSD method.

A Y-based precursor solution (**Y-S1**) with Y^3+^ concentration of 0.1 M was prepared as follows. First, 3.14 g (8 mmol) of [Y(H_2_O)_2_Lact_3_] (**3-Y**) was dispersed in several milliliters of ^i^PrOH. DETA (13 mL, 120 mmol) was added and the reaction mixture was diluted with ^i^PrOH to reach a total volume of 80 mL. The reaction mixture was stirred upon heating to ca. 90 °C until the solid reagent completely dissolved. Residual insoluble admixtures were filtered off, and the resulting clear solution was used for the deposition of thin films. La-based precursor solutions (**La-S1** and **La-S2**) were prepared in an analogous manner. The concentrations of La^3+^ ions were 0.1 M and 0.05 M for **La-S1** and **La-S2**, respectively. Additionally, the La:DETA ratio was different for these solutions, at 15:1 for **La-S1** and 13:1 for **La-S2**.

The tape substrate was submerged into the bath with the precursor solution and pulled out in the vertical direction at a rate of 1 mm/s and then passed through two tube furnaces for drying (100 °C, air atmosphere) and combustion (500 °C, ozonized air atmosphere, gas flow 5 L/min, ozone concentration 0.6 mg/L) to yield the required thin films. **Y-F1** and **La-F1** films were produced from the **Y-S1** and **La-S1** solutions, respectively, through a single deposition cycle onto as-rolled hastelloy substrates, while **La-F2** was deposited from the **La-S2** solution through seven cycles onto an electropolished hastelloy substrate. The detailed scheme of the laboratory-made MOCSD apparatus used here was reported by us earlier in [62,63].

### 3.3. X-ray Crystallography

Single-crystal XRD data were collected on three laboratory diffractometers (Bruker AXS Inc., Madison, USA): a Bruker D8 QUEST (Photon III CMOS area detector, Mo Kα radiation, Montel optics), Bruker Smart APEX II (CCD area detector, Mo Kα radiation, graphite monochromator), and Bruker Smart APEX DUO (CCD area detector, Mo Kα radiation, graphite monochromator), as well as on the XRD1 beamline (λ = 0.70000 Å, Dectris Pilatus 2M detector, DECTRIS AG, Baden, Switzerland) of the Elettra synchrotron facility (Trieste, Italy). Diffraction datasets obtained with the laboratory diffractometers were indexed and integrated using SAINT from the SHELXTL PLUS package [64,65,66], whereas the synchrotron diffraction data were processed using CrysAlisPRO v.40.67a [67] software. Crystal structures were solved by direct methods (SHELXS) and refined anisotropically for all non-H atoms with the full-matrix *F*^2^ least-squares technique (SHELXL). Hydrogen atoms of water molecules and hydroxyl groups were located from the difference Fourier maps and refined with soft restraints on O–H distances and H–O–H angles. All other H atoms were placed in geometrically calculated positions and refined in a riding model. Absorption correction was applied using SADABS v. 2016/2 [68] and SCALE3 ABSPACK v.1.0.4 [69]. Further details on the data collection and refinement parameters are summarized in Table S1.

The crystal structures of **3-Sm** and **3-Y** were determined from powder XRD data by Rietveld refinement. PXRD data were collected on a Rigaku SmartLab (Rigaku, Tokyo, Japan) diffractometer (9 kW rotating anode, Cu Kα radiation, secondary graphite monochromator) operated in symmetrical reflection θ–θ mode. The powder sample was placed into a side-loaded sample holder, which was rotated along the φ-axis during the measurements in order to avoid texturing effects and to improve the statistics. Structures were solved using the initial model prepared from the **4-Sm** homologue by omitting free water molecules and distorting the unit cell, followed by the full-profile Rietveld method in JANA2006 [70] with soft restraints on bond distances and valence angles within Lact-anions as well as on Ln–O bond distances. Positions of the H-atoms of water molecules were determined geometrically based on the analysis of H-bonds, while other H atoms were placed in idealized positions. All atoms were refined isotropically with Uiso(Ln), a common Uiso(C) for all C-atoms, the Uiso(O_Lact_) of all O-atoms of Lact-anions, and Uiso(O_W_) for the O atoms of H_2_O molecules. All H-atoms were refined in a riding model with fixed isotropic thermal parameters. PXRD patterns were fitted with a nine-term Legendre polynomial background and a five-term pseudo-Voigt peak shape function, with asymmetry corrected by axial divergence.

Continuous Shape Measures (CShM) analysis of RE coordination polyhedra in the crystal structures of **1-Ln**–**4-Ln** was carried out using Shape v.2.1 software [55].

CCDC 2277672–2277680 contain the supplementary crystallographic data for this paper. These data can be obtained free of charge via http://www.ccdc.cam.ac.uk/conts/retrieving.html (accessed on 30 June 2023) or from the CCDC, 12 Union Road, Cambridge CB2 1EZ, UK; Fax: +44 1223 336033; E-mail: deposit@ccdc.cam.ac.uk.

## 4. Conclusions

In general, rare-earth carboxylates exhibit an extreme structural diversity; introduction of ancillary functional groups into the structure of anionic ligands (e.g., OH-groups) may lead to novel metal–ligand binding modes, yielding unusual molecular and supramolecular motifs. In the present work, this feature has been demonstrated for rare-earth lactates with crystal structures of four different types; these have been systematically studied throughout the whole rare-earth series. The main feature of all structures revealed here is the presence of five-membered chelate rings formed by rare-earth cations and lactate ligands. Unlike non-substituted rare-earth carboxylates (e.g., acetates), which are prone to forming oligonuclear and polymeric complexes through bridging and chelating-bridging coordination, metal–lactate chelate rings favor the formation of mononuclear complexes for medium- and small-radius rare-earth cations (Sm–Lu, Y). Nevertheless, additional chelating-bridging coordination of lactate ligands, which yields binuclear or polymeric species, is possible if the coordination environment of the metal center is not fully saturated by chelate rings (e.g., for La and Ce–Nd lactates). Interestingly, mononuclear Sm–Lu and Y lactate hydrates have been found to exhibit polytypism, i.e., polymorphism implying different arrangements of hydrogen-bonded layers with the virtually identical configuration of a single layer. Regarding the practical application of rare-earth lactates, these have been proven to be efficient precursors for the chemical solution deposition of rare-earth oxide thin films, as demonstrated for the example of Y_2_O_3_ and La_2_O_3_ coatings. In addition to their high smoothness, thin films of La_2_O_3_ are also prospective high-k materials (κ~27) for semiconductor electronics. Furthermore, molecular lactate complexes exist for many transition metals, solutions of which are expected to be compatible with those of rare-earth lactates. This makes it possible to use metal lactates for the fabrication of 3d–4f heterometallic oxide nanomaterials (rare-earth nickelates, ferrites, manganites, etc.), which is the primary goal of our further research.

## Data Availability

The primary data presented in this study are available on request from the corresponding author.

## Supplementary Materials

The following supporting information can be downloaded at: https://www.mdpi.com/article/10.3390/molecules28155896/s1, Figure S1: TG-DTG curves for [Ln(H_2_O)_2_Lact_3_]_∞_ (**1-Ln**; Ln = La, Ce, Pr) and [Ln_2_(H_2_O)_5_Lact_6_]∙H_2_O (**2-Ln**; Ln = La, Ce, Pr, Nd); Figure S2: TG-DTG curves for [Ln(H_2_O)_2_Lact_3_] (**3-Ln**; Ln = Sm–Ho; part 1); Figure S3: TG-DTG curves for [Ln(H_2_O)_2_Lact_3_] (**3-Ln**; Ln = Er–Lu, Y; part 2); Figure S4: TG-DTG curves for [Ln(H_2_O)_2_Lact_3_]∙H_2_O (**4-Ln**; Ln = Sm–Gd, Y); Table S1: Crystal and refinement data for **1-Ln**–**4-Ln**; Table S2: Selected interatomic distances for the crystal structures of **1-Ln** and **2-Ln** estimated from single-crystal and powder XRD data; Table S3: Selected interatomic distances for the crystal structures of **3-Ln** and **4-Ln** estimated from single-crystal and powder XRD data; Table S4: Continuous Shape Measures (CShM) analysis of RE coordination polyhedra in the crystal structures of **1-Ln**–**4-Ln**; Figure S5: Room-temperature PXRD data for **3-Sm**: the experimental pattern, Rietveld refinement fit, difference profile, and positions of Bragg peaks; Figure S6: Room-temperature PXRD data for **3-Y**: the experimental pattern, Rietveld refinement fit, difference profile, and positions of Bragg peaks; Figure S7: Comparison of the layer arrangement for **3-Gd** and **5-Y**; Figure S8: Sections of VT-PXRD color maps (Mo Kα radiation) for **2-Pr** and **4-Gd** lactates in comparison with the respective theoretical PXRD patterns; Figure S9: AFM topography (5 × 5 μm^2^ scans) of the Hastelloy substrates (as-rolled and electropolished tapes); Figure S10: θ–θ XRD scan for the **Y-F1** thin film; Figure S11: AFM topography (5 × 5 μm^2^ scan) of the **La-F2** film on the electropolished Hastelloy HC276 substrate.

## Sample Availability

Samples of compounds **1-Ln**–**4-Ln** are available from the authors.
