# Supplementary material for: Diverse Coordination Chemistry of the Whole Series Rare-Earth L-Lactates: Synthetic Features, Crystal Structure, and Application in Chemical Solution Deposition of Ln2O3 Thin Films
Source: Molecules. 2023 Aug 5;28(15):5896. doi: 10.3390/molecules28155896 (PMC10421212; doi:10.3390/molecules28155896)
Supplement: Supplementary file 1 [file molecules-28-05896-s001.zip › molecules-2507762-supplementary.pdf]

Supplementary Materials

Diverse Coordination Chemistry of the Whole Series Rare-Earth L-Lactates: Synthetic Features, Crystal Structure, and Application in Chemical Solution Deposition of Ln_2O_3 Thin Films

Ruslan Gashigullin [1], Mikhail Kendin [1,2], Irina Martynova [2] and Dmitry Tsymbarenko [2,*]

[1] Department of Materials Science, Lomonosov Moscow State University, Moscow 119991, Russia;
[2] Department of Chemistry, Lomonosov Moscow State University, Moscow 119991, Russia;
* Correspondence: tsymbarenko@gmail.com

Abstract: Rare-earth (RE, Ln) carboxylates are widely studied as precursors of RE oxide-based nanomaterials; however, no systematic studies of RE L-lactates (HLact = 2-hydroxypropanoic acid) have been reported so far. In the present work, a profound structural investigation of RE L-lactates has been carried out. A family of RE lactate complexes of the general formula $LnLact_3 \cdot nH_2O$ (Ln = La, Ce–Nd, Sm–Lu, Y; n = 2–3) have been synthesized and characterized by CHN, TGA, and FTIR as well as by powder and single-crystal XRD methods. The existence of four novel structural types (**1-Ln** – **4-Ln**) has been revealed. Compounds of the **1-Ln** type (Ln = La, Ce, Pr) exhibit a chain polymeric structure, whereas **2-Ln** – **4-Ln** compounds are molecular crystals consisting of dimeric (**2-Ln**; Ln = La, Ce–Nd) or monomeric (**3-Ln** – Ln = Sm–Lu, Y; **4-Ln** – Ln = Sm–Gd, Y) species. Crystal structures of **1-Ln** – **4-Ln** are discussed in terms of coordination geometry and supramolecular arrangement. Solutions of yttrium and lanthanum lactates with diethylenetriamine have been applied for the chemical deposition of Y_2O_3 and La_2O_3 thin films.

Keywords: lanthanide; coordination compound; coordination polymer; 2-hydroxypropanoate; lactic acid; X-ray diffraction; crystal structure; thin film; chemical solution deposition

---

Table of Contents

Section S1. TGA Study of RE Lactates **S3**

Figure S1. TG-DTG curves for $[Ln(H_2O)_2Lact_3]_\infty$ (**1-Ln**; Ln = La, Ce, Pr) and $[Ln_2(H_2O)_5Lact_6]\cdot H_2O$ (**2-Ln**; Ln = La, Ce, Pr, Nd) S3
Figure S2. TG-DTG curves for $[Ln(H_2O)_2Lact_3]$ (**3-Ln**; Ln = Sm–Ho; part 1) S4
Figure S3. TG-DTG curves for $[Ln(H_2O)_2Lact_3]$ (**3-Ln**; Ln = Er–Lu, Y; part 2) S5
Figure S4. TG-DTG curves for $[Ln(H_2O)_2Lact_3]\cdot H_2O$ (**4-Ln**; Ln = Sm–Gd, Y) S6

Section S2. Single-crystal and Powder XRD study RE lactates **S7**

Table S1. Crystal and refinement data for **1-Ln – 4-Ln** S7
Table S2. Selected interatomic distances for the crystal structures of **1-Ln** and **2-Ln** estimated from single-crystal and powder XRD data S8
Table S3. Selected interatomic distances for the crystal structures of **3-Ln** and **4-Ln** estimated from single-crystal and powder XRD data S9
Table S4. Continuous Shape Measures (CShM) analysis of RE coordination polyhedra in the crystal structures of **1-Ln – 4-Ln** S10
Figure S5. Room-temperature PXRD data for **3-Sm**: the experimental pattern, Rietveld refinement fit, difference profile, and positions of Bragg peaks S11
Figure S6. Room-temperature PXRD data for **3-Y**: the experimental pattern, Rietveld refinement fit, difference profile, and positions of Bragg peaks S12
Figure S7. Comparison of the layer arrangement for **3-Gd** and **5-Y** S13
Figure S8. Sections of VT-PXRD color maps (Mo Kα radiation) for **2-Pr** and **4-Gd** lactates in comparison with the respective theoretical PXRD patterns S14

Section S3. Characterization of thin films **S15**

Figure S9. AFM topography (5 × 5 μm² scans) of the Hastelloy substrates (as-rolled and electropolished tapes) S15
Figure S10. θ–θ XRD scan for the **Y-F1** thin film S16
Figure S11. AFM topography (5 × 5 μm² scan) of the **La-F2** film on the electropolished Hastelloy HC276 substrate S17

References **S18**

Section S1. TGA study of RE lactates

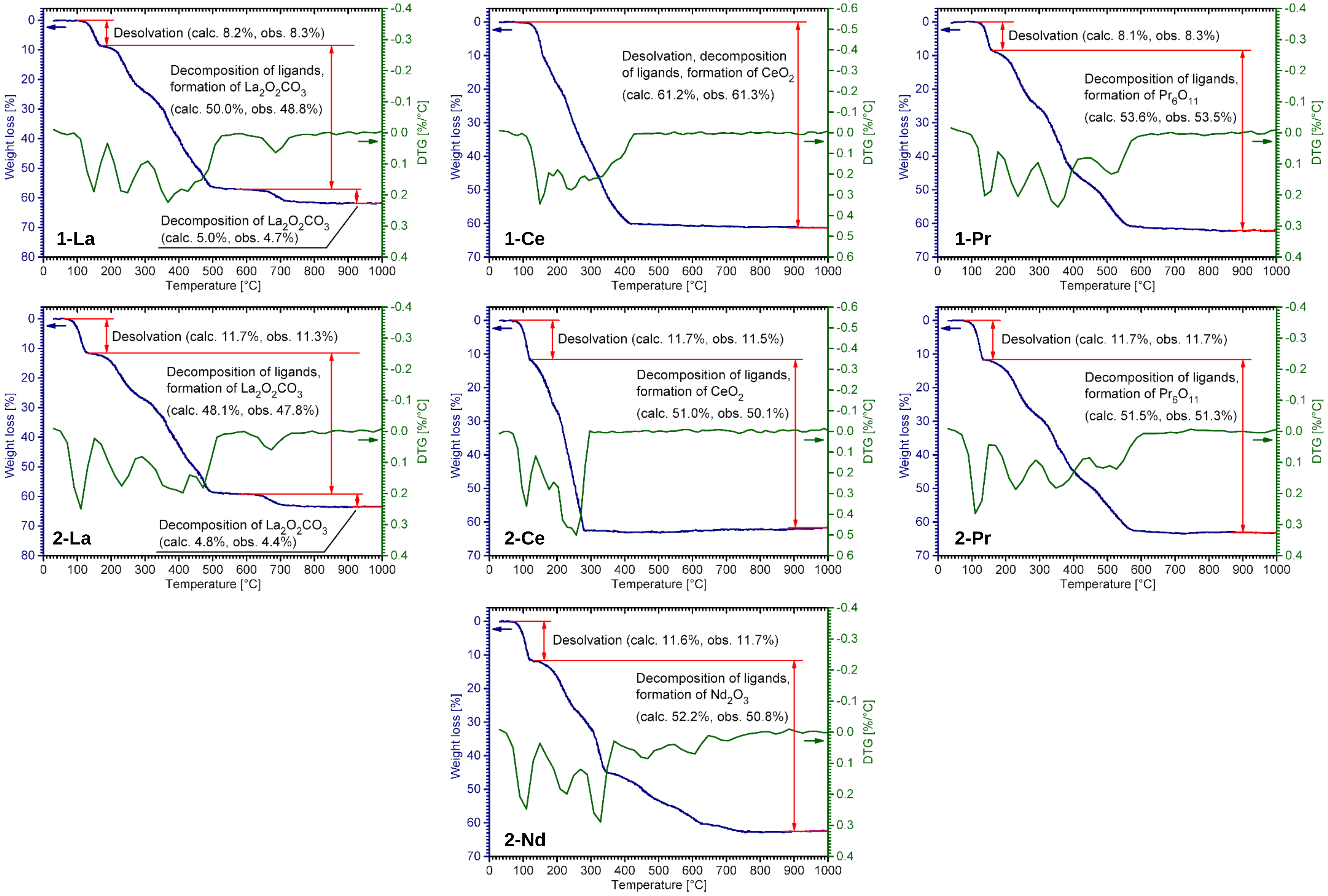

Figure S1. TG-DTG curves for $[Ln(H_2O)_2Lact_3]_\infty$ (**1-Ln**; Ln = La, Ce, Pr) and $[Ln_2(H_2O)_5Lact_6]\cdot H_2O$ (**2-Ln**; Ln = La, Ce, Pr, Nd).

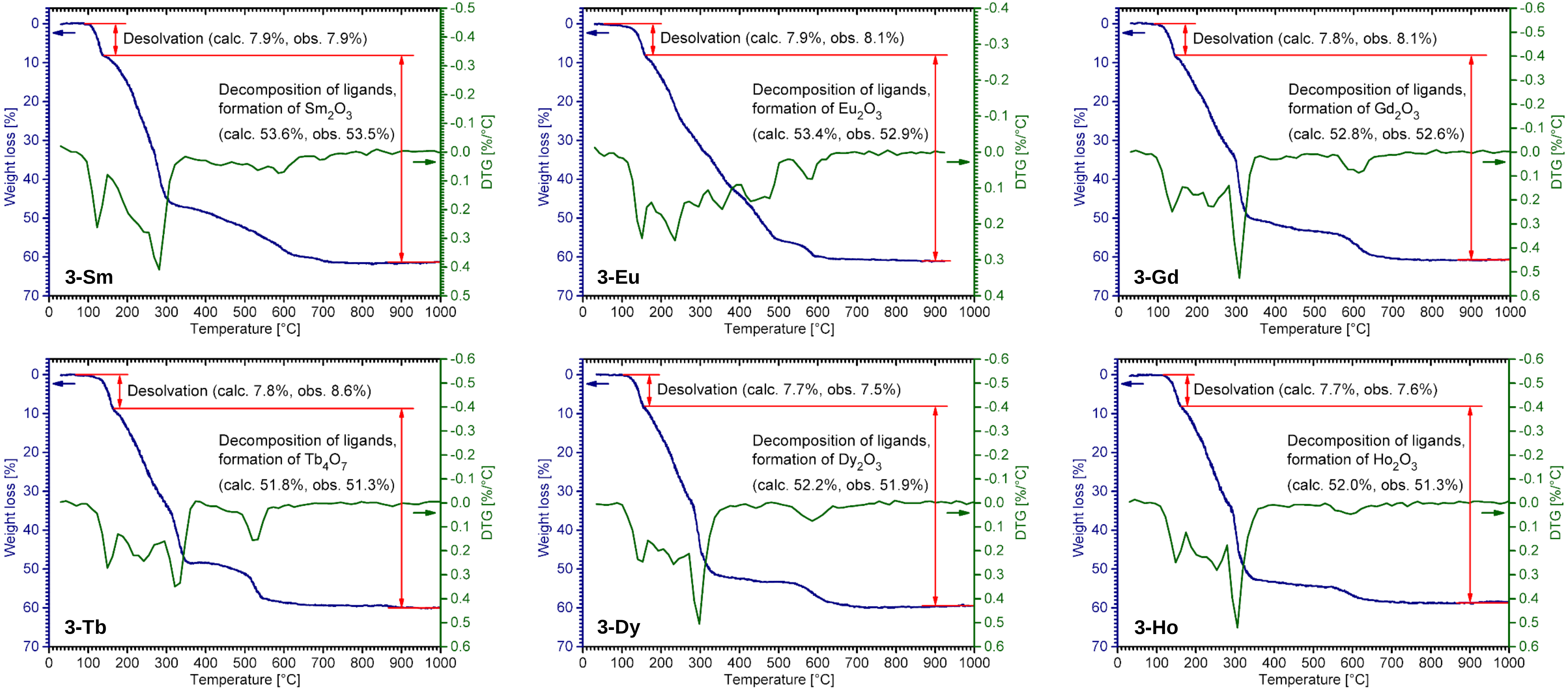

Figure S2. TG-DTG curves for [Ln(H_2O)$_2$Lact$_3$] (**3-Ln**; Ln = Sm–Ho; part 1).

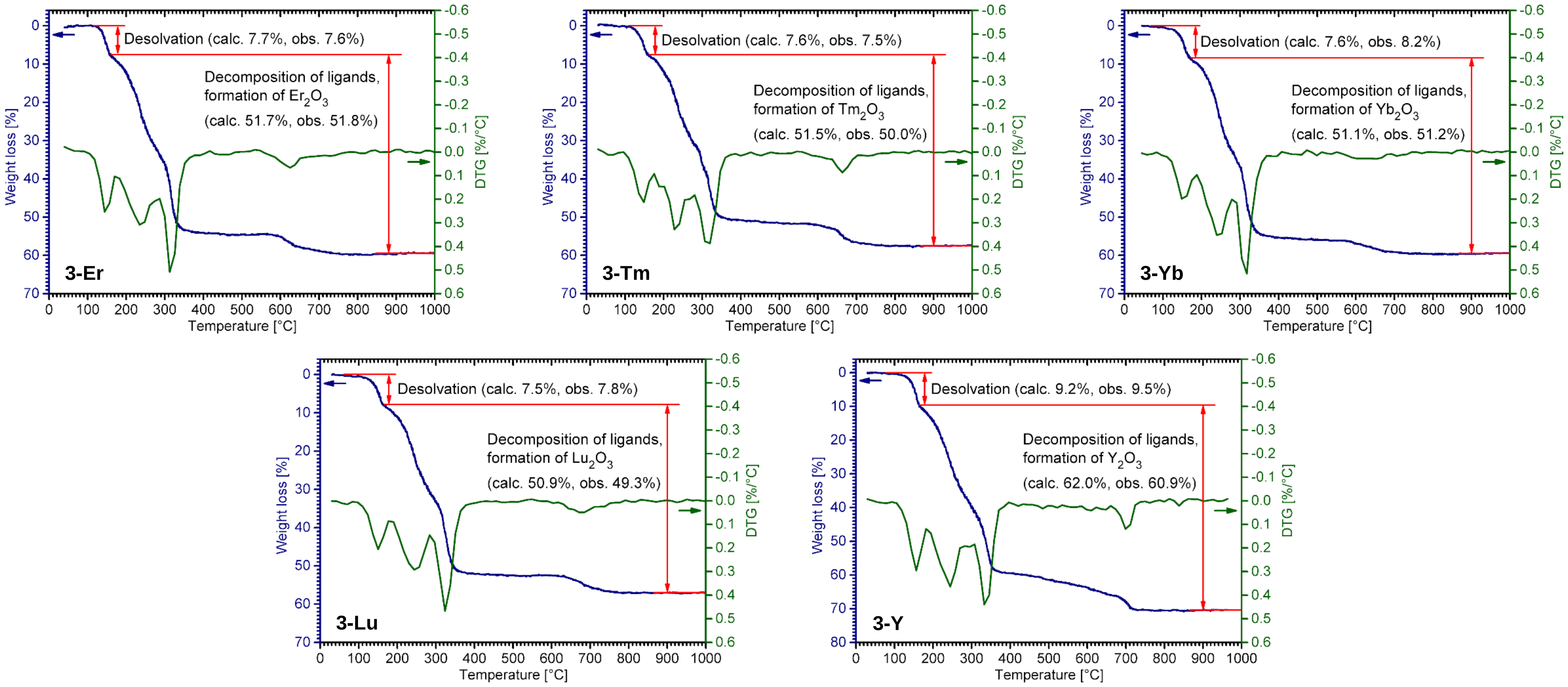

Figure S3. TG-DTG curves for [Ln(H_2O)$_2$Lact$_3$] (**3-Ln**; Ln = Er–Lu, Y; part 2).

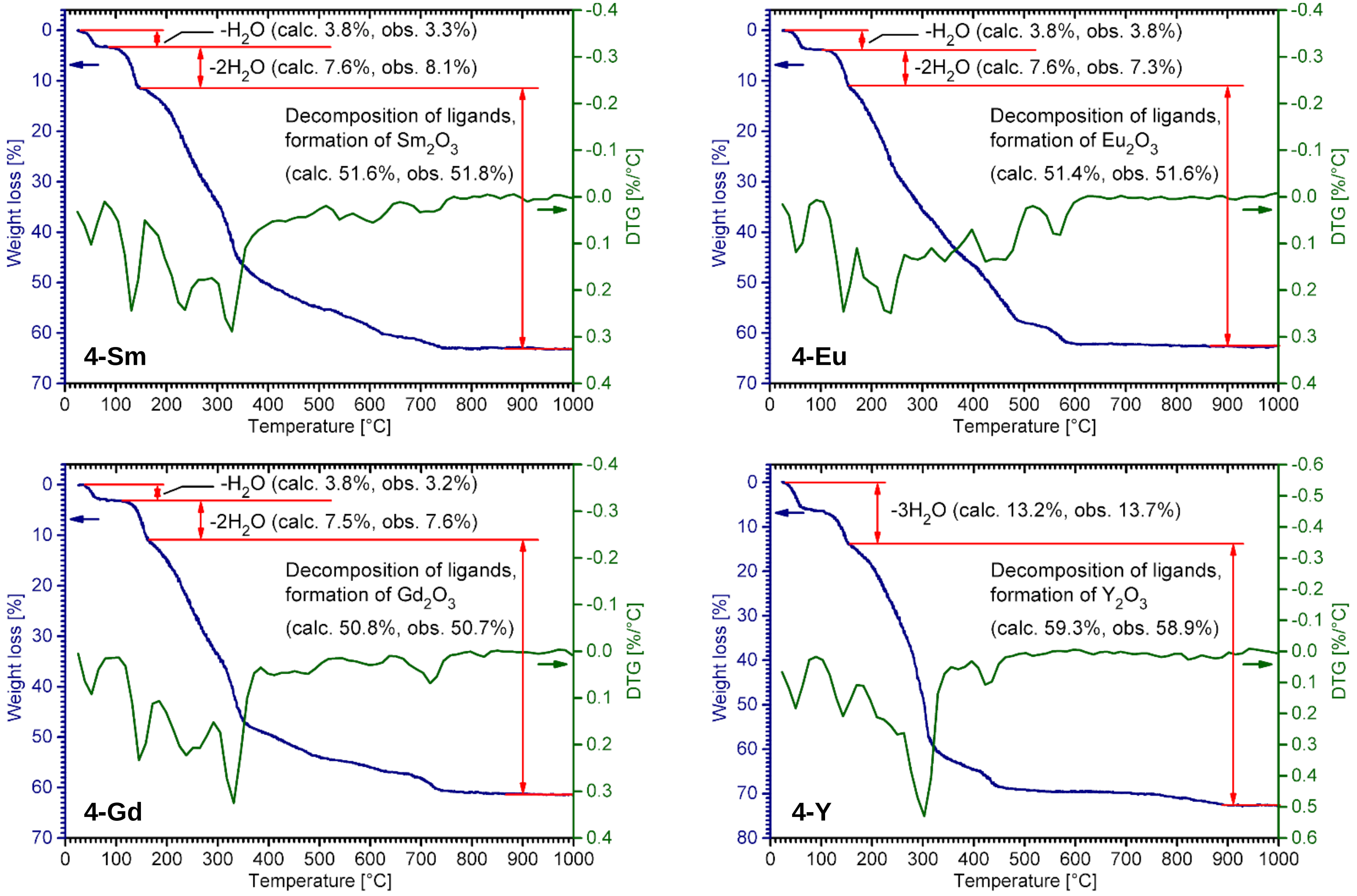

Figure S4. TG-DTG curves for [Ln(H_2O)$_2$Lact$_3$]·H_2O (**4-Ln**; Ln = Sm–Gd, Y).

Section S2. Single-crystal and powder XRD study of RE lactates

Table S1. Crystal and refinement data for **1-Ln – 4-Ln**.

	**$[Ln(H_2O)_2Lact_3]_\infty$ (1-Ln)**		**$[Ln_2(H_2O)_5Lact_6]\cdot H_2O$ (2-Ln)**			**$[Ln(H_2O)_2Lact_3]$ (3-Ln) and $[Ln(H_2O)_2Lact_3]\cdot H_2O$ (4-Ln)**			
	**1-La**	**1-Pr**	**2-La**	**2-Ce**	**2-Pr**	**3-Sm**	**3-Gd**	**3-Y**	**4-Sm**
Formula	$C_9H_{19}LaO_{11}$	$C_9H_{19}O_{11}Pr$	$C_{18}H_{42}La_2O_{24}$	$C_{18}H_{42}Ce_2O_{24}$	$C_{18}H_{42}O_{24}Pr_2$	$C_9H_{19}O_{11}Sm$	$C_9H_{19}GdO_{11}$	$C_9H_{19}O_{11}Y$	$C_9H_{21}O_{12}Sm$
Formula weight ($g\cdot mol^{-1}$)	442.15	444.15	920.34	922.76	924.33	453.60	460.49	392.10	471.61
Diffractometer	Bruker Smart APEX II	Bruker D8 QUEST	Bruker Smart APEX II	Bruker Smart APEX II	Bruker D8 QUEST	Rigaku SmartLab	XRD1 beamline – Elettra	Rigaku SmartLab	Bruker Smart APEX DUO
Radiation	Mo $K\alpha$	Mo $K\alpha$	Mo $K\alpha$	Mo $K\alpha$	Mo $K\alpha$	Cu $K\alpha$	λ = 0.70000 Å	Cu $K\alpha$	Mo $K\alpha$
Data collection method	ω-scans	ω-scans	ω-scans	ω-scans	ω-scans	θ–θ-scan	φ-scans	θ–θ-scan	ω-scans
Temperature (K)	120(2)	100(2)	120(2)	120(2)	100(2)	293(2)	100(2)	293(2)	120(2)
Crystal system	Orthorhombic	Orthorhombic	Triclinic	Triclinic	Triclinic	Orthorhombic	Orthorhombic	Orthorhombic	Orthorhombic
Space group	$P2_12_12_1$	$P2_12_12_1$	$P1$	$P1$	$P1$	$P2_12_12_1$	$P2_12_12_1$	$P2_12_12_1$	$P2_12_12_1$
a (Å)	7.7106(6)	7.6358(2)	9.1234(4)	9.0958(4)	9.0442(4)	5.77537(17)	5.7542(2)	5.7648(4)	5.8257(5)
b (Å)	10.3304(8)	10.2649(3)	9.9465(4)	9.9170(4)	9.8678(4)	10.87019(19)	10.7147(3)	10.7839(3)	13.3765(12)
c (Å)	17.9109(14)	17.8049(5)	11.0264(4)	11.0009(4)	10.9410(4)	25.5881(4)	25.3556(8)	25.3685(6)	20.6750(19)
α (^{0})	90	90	63.276(2)	63.2393(5)	63.2426(7)	90	90	90	90
β (^{0})	90	90	65.574(3)	65.5623(5)	65.5864(8)	90	90	90	90
γ (^{0})	90	90	88.5330(10)	88.6254(6)	88.6283(9)	90	90	90	90
V ($Å^3$)	1426.67(19)	1395.56(7)	797.75(6)	790.42(6)	777.98(6)	1606.41(6)	1563.28(9)	1577.09(12)	1611.2(2)
Z	4	4	1	1	1	4	4	4	4
Color, habit	Colorless, block	Pale green, block	Colorless, plate	Colorless, block	Pale green, block	White, powder flat sheet	Colorless, needle	White, powder flat sheet	Colorless, prism
Crystal/sample dimensions (mm)	0.16×0.13×0.11	0.289×0.204×0.173	0.32×0.24×0.07	0.35×0.22×0.18	0.12×0.07×0.06	20×20×0.5	0.15×0.03×0.01	20×20×0.5	0.35×0.22×0.21
D_{calc} ($g\cdot cm^{-3}$)	2.059	2.114	1.916	1.939	1.973	1.8755	1.957	1.6516	1.944
μ (mm^{-1})	3.048	3.546	2.734	2.937	3.189	27.93	4.138	27.93	3.701
Unique reflections (R_{int})	4147 (0.0450)	4053 (0.0346)	8799 (0.0136)	8707 (0.0147)	12492 (0.0155)	839 (–)	3406 (0.0607)	980 (–)	5994 (0.0319)
Observed reflections [$I > 2\sigma(I)$]	3940	3872	8637	8574	12354	809	3116	980	5835
Parameters, restraints	193, 0	199, 7	403, 3	404, 3	416, 21	85, 45	198, 49	82, 45	209, 15
R_1 [$I > 2\sigma(I)$], ωR_2	0.0256, 0.0576	0.0152, 0.0308	0.0151, 0.0357	0.0160, 0.0372	0.0148, 0.0361	–	0.0429, 0.1419	–	0.0250, 0.0555
R_{Bragg}, R_p, ωR_p	–	–	–	–	–	0.0427, 0.0636, 0.0811	–	0.0269, 0.0584, 0.0838	–
Goodness-of-fit [1]	0.974	1.041	1.055	1.042	0.977	1.49	1.211	3.73	1.071
Absorption correction	SADABS	SADABS	SADABS	SADABS	SADABS	Not required	ABSPACK	Not required	SADABS
T_{min}, T_{max}	0.7179, 0.9222	0.4811, 0.7182	0.5905, 0.7056	0.5227, 0.6032	0.6129, 0.7468	–	0.8739, 0.9789	–	0.3529, 0.5197
ρ_{min}, ρ_{max}	–1.048, 1.209	–0.395, 0.352	–0.421, 0.618	–0.522, 0.692	–0.816, 0.711	–1.26, 0.53	–2.649, 3.742	–0.29, 0.35	–1.765, 1.270

[1] Goodness-of-fit is calculated on F^2 for single-crystal XRD data (all experiments except **3-Sm** and **3-Y**) and on the overall pattern intensities for PXRD data (**3-Sm** and **3-Y**).

Table S2. Selected interatomic distances for the crystal structures of **1-Ln** and **2-Ln** estimated from single-crystal and powder XRD data. [1]

**$[Ln(H_2O)_2Lact_3]_\infty$ (1-Ln)**			**$[Ln_2(H_2O)_5Lact_6]\cdot H_2O$ (2-Ln)**			
	**1-La**	**1-Pr**		**2-La**	**2-Ce**	**2-Pr**
Bond	d (Å)		Bond	d (Å)		
Ln1–O1	2.547(2)	2.509(2)	Ln1–O1	2.5658(15)	2.5434(16)	2.5226(16)
			Ln1–O3	2.5243(15)	2.5046(17)	2.4798(16)
Ln1–O3	2.610(2)	2.5670(18)	Ln1–O4	2.5218(15)	2.4948(17)	2.4677(16)
			Ln1–O6	2.6264(15)	2.6106(17)	2.5819(16)
Ln1–O4	2.569(2)	2.527(2)	Ln1–O7	2.5013(15)	2.4816(16)	2.4574(16)
			Ln1–O9	2.5778(15)	2.5578(17)	2.5355(17)
Ln1–O5[i]	2.452(2)	2.422(2)	Ln1–O10	2.4744(15)	2.4502(17)	2.4255(17)
			Ln1–O19	2.5647(16)	2.5452(17)	2.5251(18)
Ln1–O6	2.565(2)	2.515(2)	Ln1–O20	2.5813(15)	2.5569(18)	2.5382(17)
			Ln2–O11	2.5091(15)	2.4838(16)	2.4599(16)
Ln1–O7	2.528(2)	2.480(2)	Ln2–O12	2.6143(15)	2.5943(16)	2.5718(16)
			Ln2–O13	2.5233(15)	2.4939(17)	2.4665(17)
Ln1–O9	2.608(2)	2.570(2)	Ln2–O15	2.5017(15)	2.4790(17)	2.4525(16)
			Ln2–O16	2.5131(15)	2.4941(16)	2.4690(16)
Ln1–O10	2.586(2)	2.541(2)	Ln2–O18	2.5283(15)	2.5033(16)	2.4797(16)
			Ln2–O21	2.5870(15)	2.5705(17)	2.5393(17)
Ln1–O11	2.565(2)	2.522(2)	Ln2–O22	2.5360(15)	2.5091(17)	2.4896(17)
			Ln2–O23	2.6606(16)	2.6452(17)	2.6230(18)
Symmetry codes						
(i) 1–x, –0.5+y, 1.5–z			–			

Table S3. Selected interatomic distances for the crystal structures of **3-Ln** and **4-Ln** estimated from single-crystal and powder XRD data. [1]

	$[Ln(H_2O)_2Lact_3]$ (3-Ln)			$[Ln(H_2O)_2Lact_3]\cdot H_2O$ (4-Ln)	
	**3-Sm** [1]	**3-Gd**	**3-Y** [1]	**4-Sm**	
Bond		d (Å)		Bond	d (Å)
Ln1–O1	2.366(18)	2.356(6)	2.297(9)	Sm1–O1	2.389(2)
Ln1–O3	2.400(19)	2.412(6)	2.353(9)	Sm1–O3	2.443(2)
Ln1–O4	2.376(15)	2.352(6)	2.308(8)	Sm1–O4	2.364(2)
Ln1–O6	2.451(19)	2.372(6)	2.366(9)	Sm1–O6	2.415(2)
Ln1–O7	2.401(18)	2.358(5)	2.342(9)	Sm1–O8	2.396(2)
Ln1–O9	2.457(17)	2.420(5)	2.392(7)	Sm1–O9	2.461(2)
Ln1–O10	2.397(19)	2.361(5)	2.355(18)	Sm1–O10	2.399(2)
Ln1–O11	2.444(14)	2.407(5)	2.429(12)	Sm1–O11	2.412(2)

[1] Crystal structures of **3-Sm** and **3-Y** were refined from powder XRD data; therefore, the precision on Ln–O bond distances for these structures is lower than that determined from single-crystal XRD data.

Table S4. Continuous Shape Measures (CShM) analysis [1] of RE coordination polyhedra in the crystal structures of **1-Ln – 4-Ln**. The best fitting reference shapes are highlighted in blue.

Nine-vertex polyhedra										Eight-vertex polyhedra			
	**1-La**	**1-Pr**	**2-La**		**2-Ce**		**2-Pr**			**3-Sm**	**3-Gd**	**3-Y**	**4-Sm**[2]
	**La1**	**Pr1**	**La1**	**La2**	**Ce1**	**Ce2**	**Pr1**	**Pr2**		**Sm1**	**Gd1**	**Y1**	**Sm1**
**EP-9**[1]	34.497	34.637	37.182	36.352	37.252	36.269	37.412	36.433	**OP-8**	34.262	32.622	32.224	33.000
**OPY-9**	21.541	21.728	22.209	21.801	22.211	21.807	22.347	21.772	**HPY-8**	21.477	22.570	23.612	22.401
**HBPY-9**	18.711	18.987	17.714	18.103	17.822	17.988	17.842	17.914	**HBPY-8**	13.256	15.093	12.673	15.025
**JTC-9**	13.448	13.535	15.586	14.957	15.607	15.018	15.576	15.211	**CU-8**	10.548	9.852	10.174	9.944
**JCCU-9**	8.167	8.198	9.382	10.096	9.361	10.090	9.342	10.090	**SAPR-8**	2.279	1.384	2.212	1.531
**CCU-9**	6.954	7.016	7.990	8.513	7.966	8.530	7.959	8.527	**TDD-8**	1.651	1.939	1.807	2.089
**JCSAPR-9**	2.558	2.400	2.451	2.342	2.387	2.236	2.366	2.187	**JGBF-8**	11.541	13.772	11.382	13.677
**CSAPR-9**	1.598	1.468	1.435	1.072	1.392	0.981	1.326	0.927	**JETBPY-8**	28.821	28.559	28.672	28.796
**JTCTPR-9**	2.641	2.530	2.706	3.739	2.641	3.671	2.562	3.628	**JBTPR-8**	2.997	2.392	2.829	2.336
**TCTPR-9**	1.328	1.209	0.893	1.249,	0.839	1.203	0.811	1.183	**BTPR-8**	2.279	1.574	1.954	1.518
**JTDIC-9**	10.996	11.050	10.534	11.380	10.623	11.466	10.875	11.603	**JSD-8**	4.022	4.561	3.923	4.640
**HH-9**	9.736	9.894	10.336	10.858	10.361	10.975	10.376	10.943	**TT-8**	11.287	10.674	10.763	10.773
**MFF-9**	1.956	1.887	1.382	0.948	1.364	0.927	1.344	0.915	**ETBPY-8**	23.025	24.069	23.805	24.257

[1] Reference shapes abbreviations:

Nine-vertex polyhedra: **EP-9** – D_{9h} enneagon; **OPY-9** – C_{8v} octagonal pyramid; **HBPY-9** – D_{7h} heptagonal bipyramid; **JTC-9** – C_{3v} Johnson triangular cupola J3; **JCCU-9** – C_{4v} capped cube J8; **CCU-9** – C_{4v} spherical-relaxed capped cube; **JCSAPR-9** – C_{4v} capped square antiprism J10; **CSAPR-9** – C_{4v} spherical capped square antiprism; **JTCTPR-9** – D_{3h} Tricapped trigonal prism J51; **TCTPR-9** – D_{3h} spherical tricapped trigonal prism; **JTDIC-9** – C_{3v} tridiminished icosahedron J63; **HH-9** – C_{2v} Hula-hoop; **MFF-9** – C_s Muffin.

Eight-vertex polyhedra: OP-8 – D_{8h} octagon; **HPY-8** – C_{7v} heptagonal pyramid; HBPY-8 – D_{6h} hexagonal bipyramid; **CU-8** – O_h cube; **SAPR-8** – D_{4d} square antiprism; **TDD-8** – D_{2d} triangular dodecahedron; **JGBF-8** – D_{2d} Johnson gyrobifastigium J26; **JETBPY-8** – D_{3h} Johnson elongated triangular bipyramid J14; **JBTPR-8** – C_{2v} biaugmented trigonal prism J50; **BTPR-8** – C_{2v} biaugmented trigonal prism; **JSD-8** – D_{2d} snub diphenoid J84; **TT-8** – T_d triakis tetrahedron; **ETBPY-8** – D_{3h} elongated trigonal bipyramid.

[2] The best fitting reference shape for Sm1 in the structure of **4-Sm** differs from that observed for **3-Sm** – presumably, the hydrogen bonding motif and supramolecular arrangement affect the fine distortion of the coordination environent.

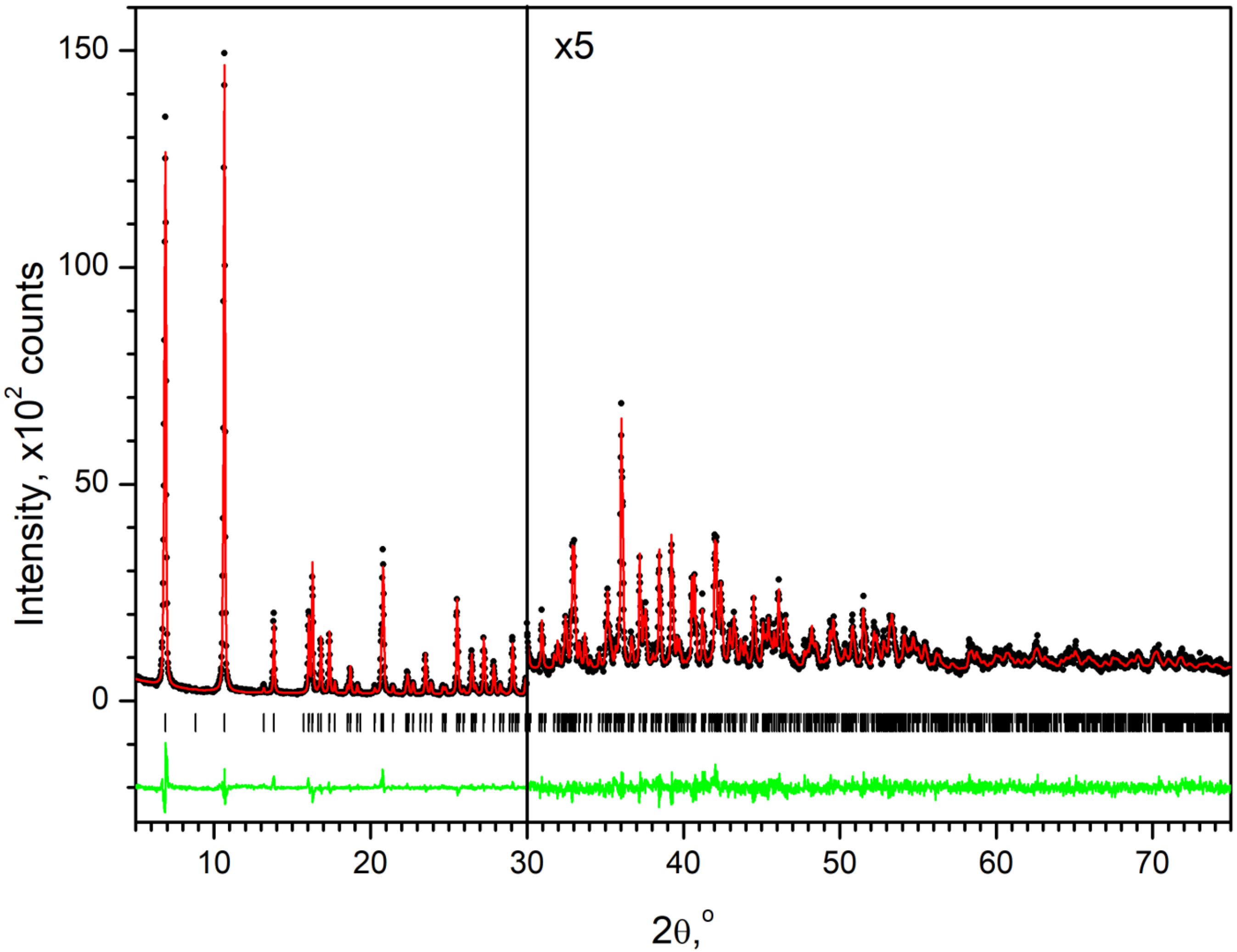

Figure S5. Room-temperature PXRD data for **3-Sm**: experimental pattern (black circles), Rietveld refinement fit (red solid line), difference profile (lower green solid line), and positions of Bragg peaks (vertical bars). The plot in the 2θ range of 30–75^0 is given at 5x magnification for clarity.The Rietveld refinement was carried out using JANA2006 software [2].

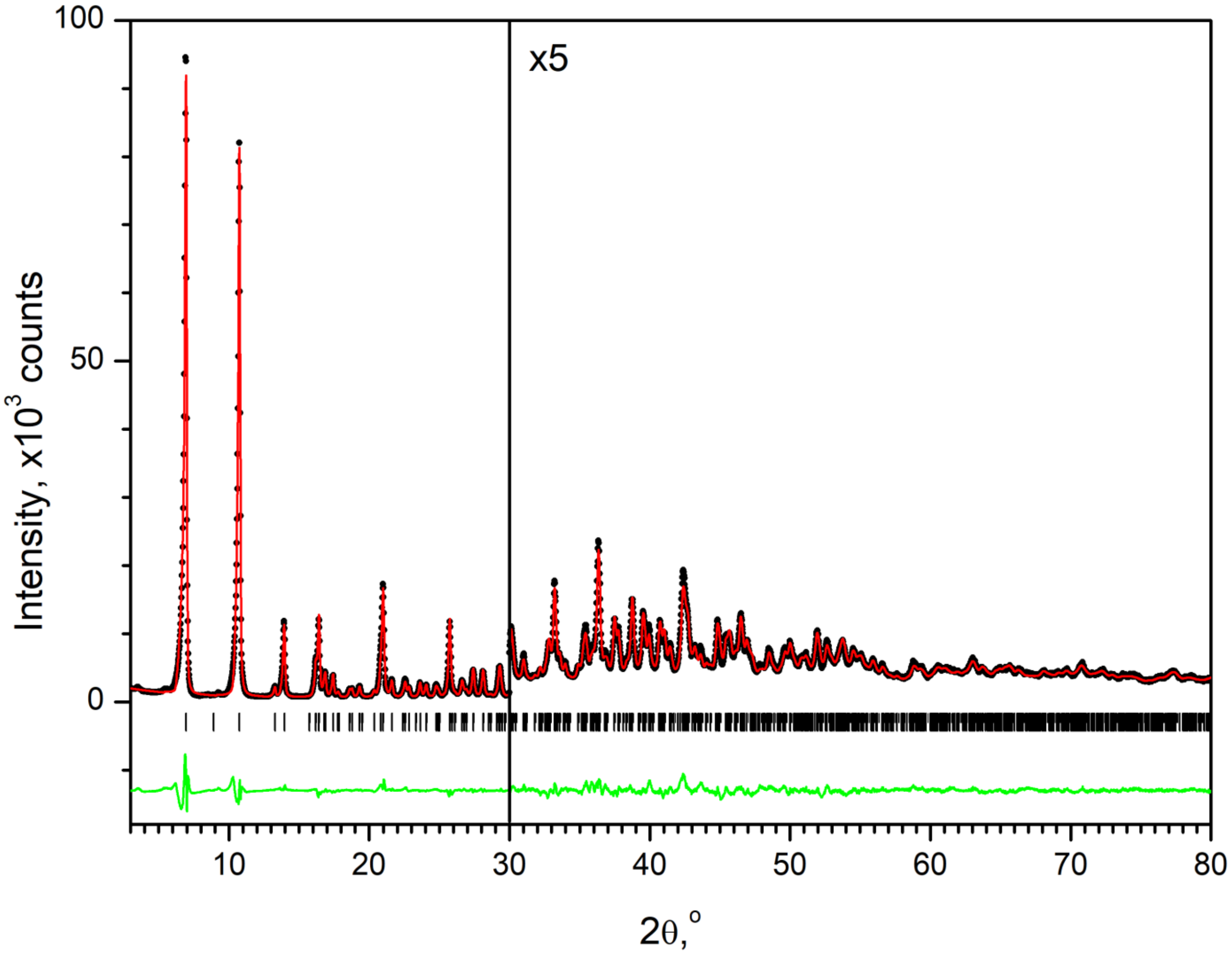

Figure S6. Room-temperature PXRD data for **3-Y**: experimental pattern (black circles), Rietveld refinement fit (red solid line), difference profile (lower green solid line), and positions of Bragg peaks (vertical bars). The plot in the 2θ range of 30–80^0 is given at 5x magnification for clarity. The Rietveld refinement was carried out using JANA2006 software [2].

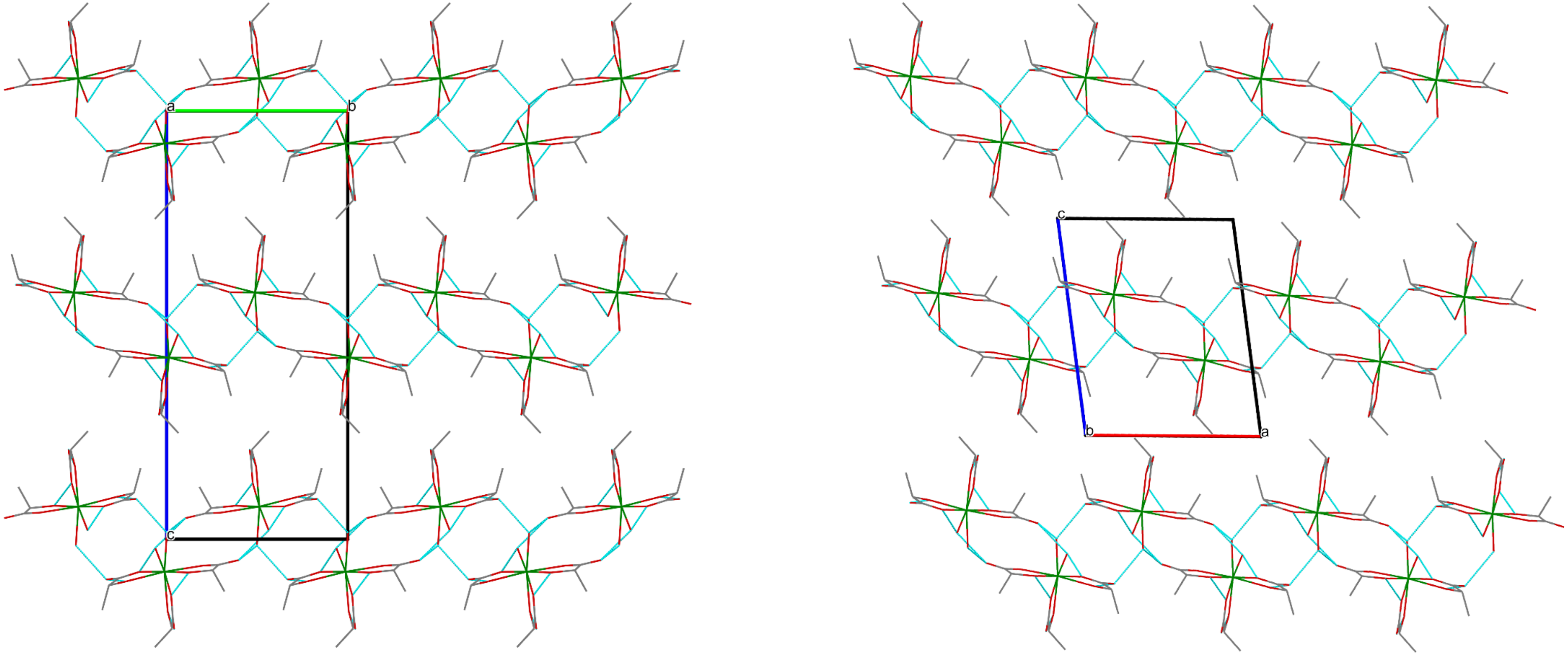

(a) **(b)**

Figure S7. Comparison of the layer arrangement for: (**a**) **3-Gd** (obtained in the present work; ABAB packing); (**b**) **5-Y** (reported by Yapryntsev *et al.* – *RSC Adv*. 2021, 11, 30195–30205, doi:10.1039/D1RA05923H; distorted AAA packing).

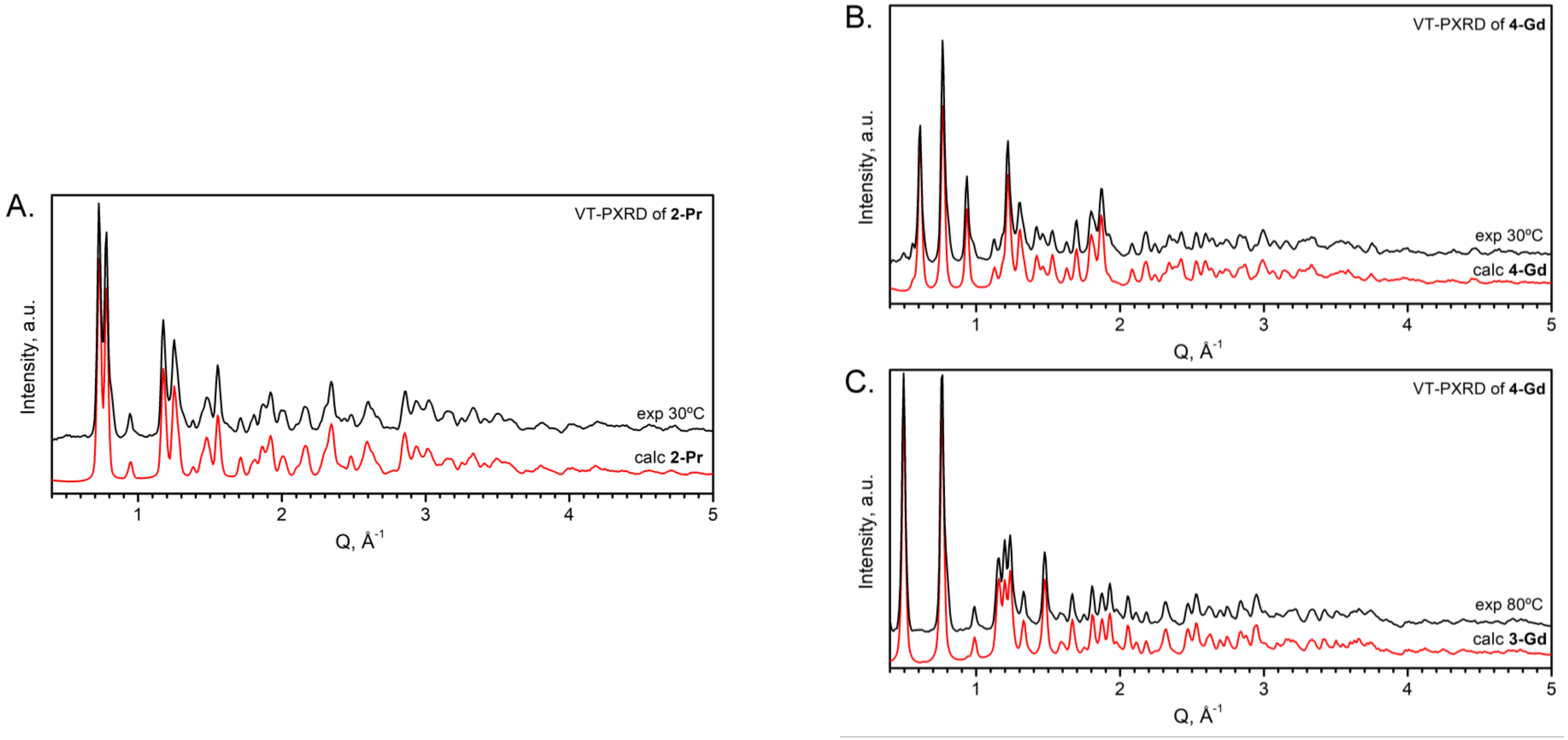

Figure S8. Sections of VT-PXRD color maps (Fig. 8, Mo Kα radiation) for **2-Pr** and **4-Gd** lactates in comparison with the respective theoretical PXRD patterns: (a) initial **2-Pr** at 30ºC; (b) initial **4-Gd** at 30ºC; (c) product of **4-Gd** heating at 80ºC (**3-Gd** phase). The theoretical profiles were calculated from the respective low-temperature (100–120 K) structure models with high-temperature (30ºC or 80ºC) unit cell parameters estimated from the full-profile refinement of experimental PXRD patterns. Q refers to the modulus of a scattering vector transfer: $Q = 4\pi \cdot sin\theta/\lambda$. The full-profile refinement was carried out using JANA2006 software [2].

Section S3. Characterization of thin films

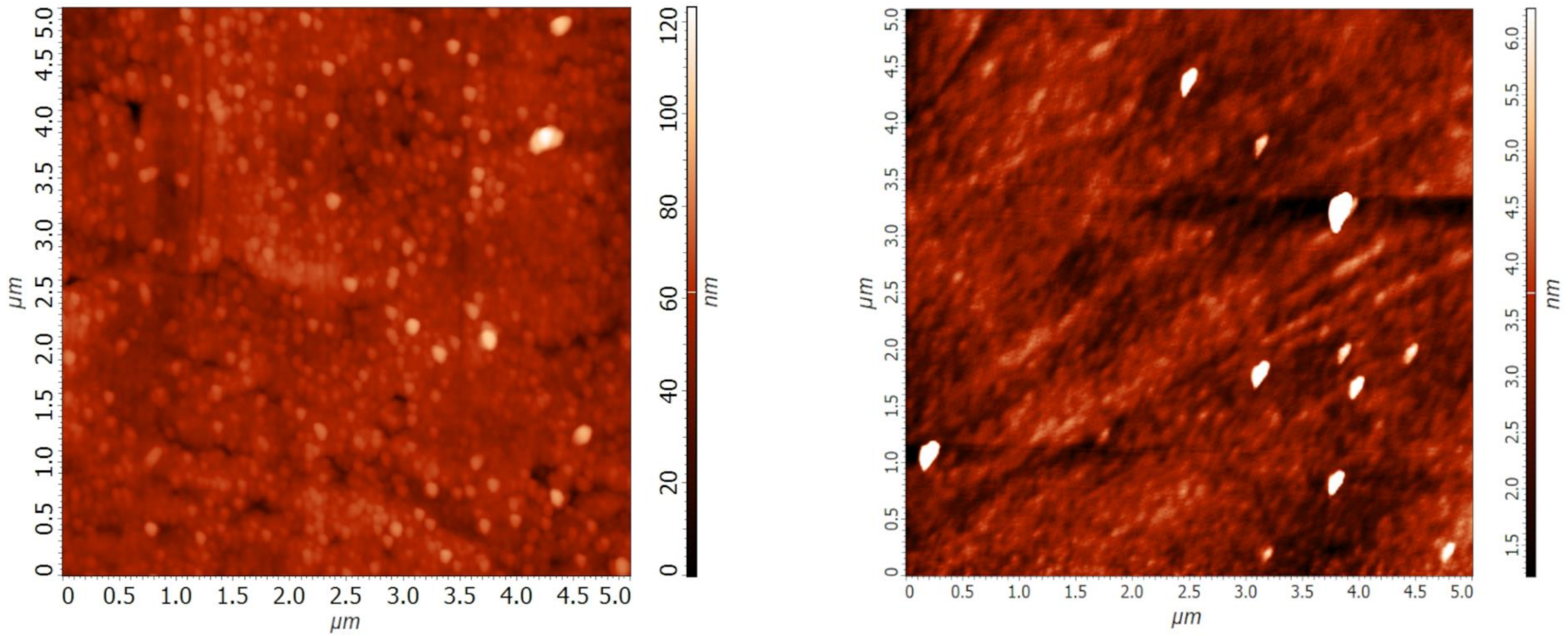

(**a**) (**b**)

Figure S9. AFM topography (5 × 5 μm² scans) of the Hastelloy HC276 substrates: (**a**) as-rolled tape; (**b**) electropolished tape.

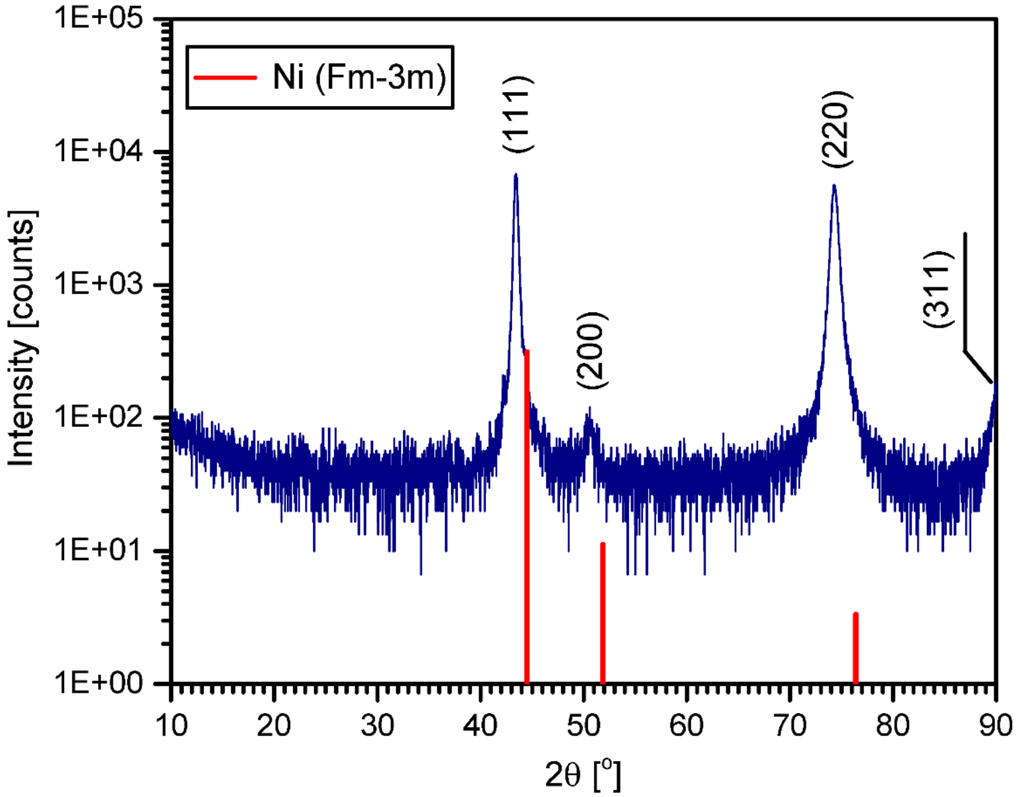

Figure S10. θ–θ XRD scan for the **Y-F1** thin film. Peaks corresponding to the polycrystalline nickel (ICDD PDF-2 card [4-850]) are depicted with red bars. Reflections from the Hastelloy HC276 tape are shifted with respect to those of polycrystalline nickel due to the presence of alloying additives in the lattice causing changes in the unit cell parameters.

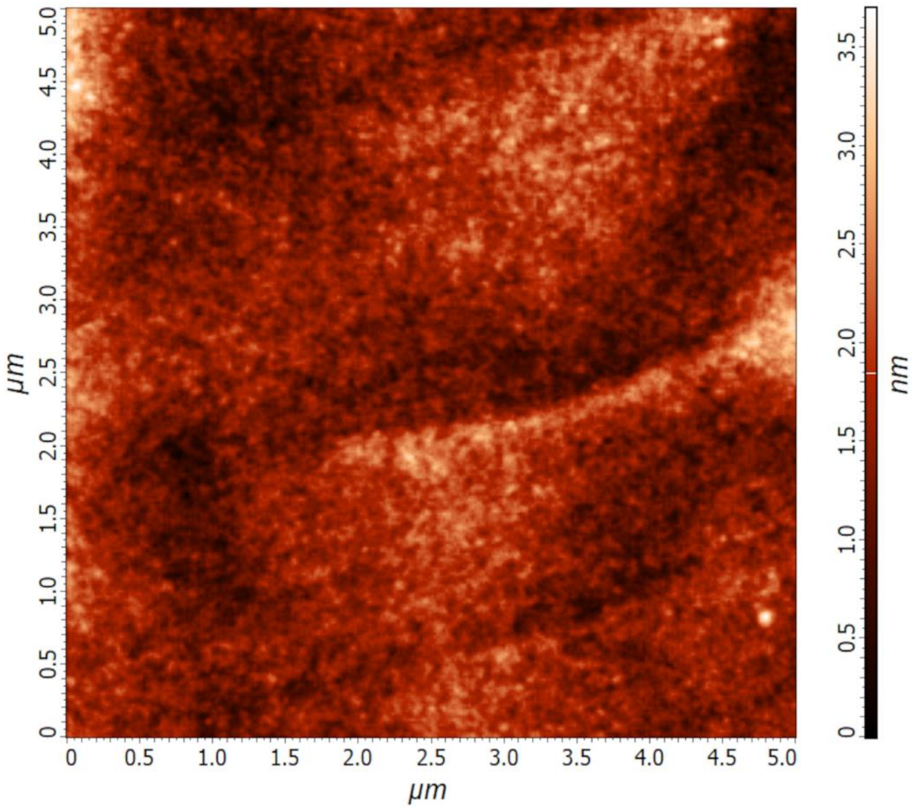

Figure S11. AFM topography (5 × 5 μm² scan) of the **La-F2** film on the electropolished Hastelloy HC276 substrate.

References

1. Casanova, D.; Llunell, M.; Alemany, P.; Alvarez, S. The Rich Stereochemistry of Eight-Vertex Polyhedra: A Continuous Shape Measures Study. *Chem. - A Eur. J.* **2005**, *11*, 1479–1494, doi:10.1002/chem.200400799.
2. Petříček, V.; Dušek, M.; Palatinus, L. Crystallographic Computing System JANA2006: General Features. *Zeitschrift für Krist. - Cryst. Mater.* **2014**, *229*, 345–352, doi:10.1515/zkri-2014-1737.
